# Polyethylene Nanocomposites for Power Cable Insulations

**DOI:** 10.3390/polym11010024

**Published:** 2018-12-24

**Authors:** Ilona Pleşa, Petru V. Noţingher, Cristina Stancu, Frank Wiesbrock, Sandra Schlögl

**Affiliations:** 1 Polymer Competence Center Leoben GmbH (PCCL), Roseggerstrasse 12, Leoben 8700, Austria; frank.wiesbrock@pccl.at (F.W.); sandra.schloegl@pccl.at (S.S.); 2 Faculty of Electrical Engineering, Electrotechnical Material Laboratory, University Politehnica of Bucharest, Splaiul Independentei 313, 060042 Bucharest, Romania; petrunot@elmat.pub.ro (P.V.N.); cstancu@elmat.pub.ro (C.S.)

**Keywords:** thermoplastic nanocomposite, polyethylene, power cable insulation, electrical property, structure-property relationship

## Abstract

This review represents a comprehensive study of nanocomposites for power cables insulations based on thermoplastic polymers such as polyethylene congeners like LDPE, HDPE and XLPE, which is complemented by original results. Particular focus lies on the structure-property relationships of nanocomposites and the materials’ design with the corresponding electrical properties. The critical factors, which contribute to the degradation or improvement of the electrical performance of such cable insulations, are discussed in detail; in particular, properties such as electrical conductivity, relative permittivity, dielectric losses, partial discharges, space charge, electrical and water tree resistance behavior and electric breakdown of such nanocomposites based on thermoplastic polymers are described and referred to the composites’ structures. This review is motivated by the fact that the development of polymer nanocomposites for power cables insulation is based on understanding more closely the aging mechanisms and the behavior of nanocomposites under operating stresses.

## 1. Introduction

**Figure 1:**
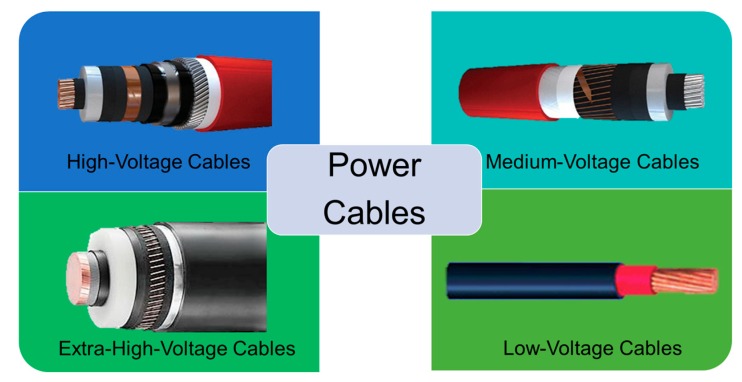
Different types of power cables used for electricity transmission and distribution.

High-voltage industry undergoes continuous development and modernization of power grid systems in order to yield reliable, cost-effective and environmentally harmless power solutions [1]. Energy power transportation across the seas and inland is targeted to be performed in particular by extruded polymer-based cables. Underground and submarine cables are used since the early stages of electricity transmission and distribution [2]. However, in regions where it is difficult or impossible to implement the overhead transmission network (i.e., densely populated zones or underwater and underground tunnels connections), high-voltage alternate current (HVAC) and high-voltage direct current (HVDC) cable networks are developed to meet the increasing capacity (Figure 1) [1]. In order to increase their levels of operating voltage and to enhance their electrical performance, it is necessary to introduce the next generation of cable insulation materials [3].

Fifty years ago, paper-insulated and oil-impregnated low-voltage (LV), medium-voltage (MV) and high voltage (HV) underground cables were used [4]. An important development took place in the 1960s, when mineral impregnation oil was mixed with small quantities of natural resin or microcrystalline petroleum wax for increasing the viscosity and avoiding the migration of impregnated oil through the cable during the heat evolvement generated by the current. Due to the significant changes in height of cables, another solution was provided by blending synthetic poly(*iso*-butylene) with microcrystalline wax using special manufacturing techniques for mass-impregnated non-draining cables above 33 kV.

**Figure 2:**
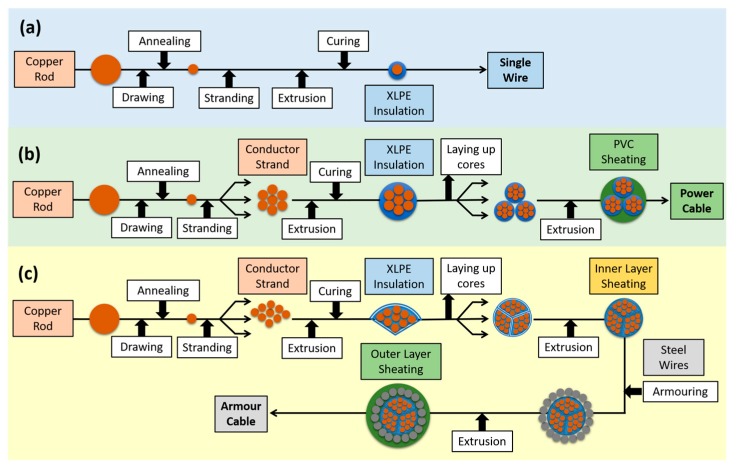
Typical process steps for the production of (**a**) wires, (**b**) power cables and (**c**) armour power cables employing thermoplast-based polymers as insulation. Redrawn and adapted from reference [5].

By using polymers such as polyethylene (PE), ethylene-propylene rubber (EPR) or ethylene-propylene-diene-monomer rubber (EPDM), it was possible to obtain high and very high voltage cables (Extra High Voltage Cables EHVC), with a low level of partial discharges, easy maintenance and remarkable longevity. The various process steps for the production of cables containing thermoplast-based insulations are provided in Figure 2.

PE was the most suitable insulation material (comprising low permittivity and high electrical breakdown strength) in the cables production in the late 1960s and early 1970s [4]. However, PE suffers from two major drawbacks: (*i*) the limitation of the maximum operating temperature, which is around 70 °C and (*ii*) the necessity to add antioxidants in order to avoid deterioration of the polymer-based insulation. Taking into consideration these aspects, a new solution was found by crosslinking of PE (yielding XLPE), which improved both, thermal resistance and ageing stability of the material due to the formation of the 3D network. The crosslinking additives such as dicumyl peroxide should not degrade the electrical performance of the crosslinked material [4]. Initially, cables based on XLPE were manufactured on continuous vulcanization lines, using steam for heating and pressurizing production stages and water under pressure for cooling. Later, it was found that the presence of steam during the crosslinking process introduced a high level of moisture in the cable insulation, resulting in the formation of microvoids in which electrical and/or water trees were developed, resulting in premature breakdown of the insulation. Hence, steam was removed from the production process. A new manufacturing process was developed involving electrical heating and pressurizing with dry nitrogen in a continuous vulcanization line. At that time, water was still used for cooling but was later eliminated from any stage of the process and generally removed as cooling method. Since the 1980s, the failure rates of XLPE cables have decreased significantly by the introduction of these new production techniques [4]. Since then, the development of XLPE for LV, HV and EHV cables with enhanced insulation quality and properties has resulted from the production of materials with fewer impurities as well as the reduction of negative effects generated by the presence of contaminants and by-products of the radical crosslinking. Other approaches involve the introduction of special tree-retardant grades of XLPE, development of colorants for the cable cores, improvement of the compatibility between XLPE and semiconductive materials for cable screens by reduction of the size of carbon black particles and the elimination of ionic contaminants [1,4].

Nowadays, XLPE cables are commonly applied but it becomes more and more difficult to improve the insulation quality, mainly due to high costs involved in the production of purer materials, which stimulates the demand for exploring other possibilities such as polymer-based nanocomposites for obtaining the targeted improvements. While paper-insulated and oil-filled cables for AC and DC applications are very easy to be used, conventional AC-XLPE insulated cables cannot be employed for DC because the electrical conductivity varies with temperature and electric field and, in particular, due to space charge accumulation [4]. For underground power cables insulation, other copolymers of ethylene and propylene (EPR) and terpolymers of ethylene, propylene and a diene component (EPDM) are typically applied [6]. They are highly filled and opaque elastomers due to their chemical and physical properties (e.g., sensitivity to heat, oxidation, ozone and weather, insolubility in many polar solvents, etc.). EPR and EPDM are flexible even at low temperatures (amorphous forms of EPR) and exhibit a certain level of tree retardancy, however at the drawback of some electrical properties (i.e., higher dissipation factor) [6].

Today, on-going research activities aim at the application of new polymer materials with or without nanoparticles (e.g., LDPE/metal oxides nanocomposites containing additional voltage stabilizers), processed by modern methods, which are very promising materials for the future of cables insulation for DC and AC applications.

This publication provides a review of the most important thermoplast-based nanocomposites (i.e., based on LDPE, HDPE, XLPE) used as power cables insulation, starting with their chemical structure, addressing their electrical properties and establishing structure-property-relationships. Particularly for cables insulations based on nanocomposites, the critical factors, which are contributing to the degradation or improvement of the electrical performance under stress, are discussed, with a particular focus on the influence of nanofillers and additives on the electrical properties of the cables’ insulation.

## 2. Critical Challenges of Polymer-Based Nanocomposites in Industrial Applications

**Figure 3:**
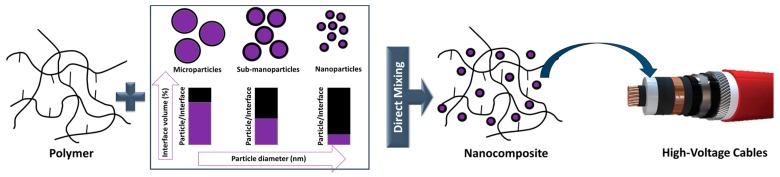
Schematic representation of polymers nanocomposites used for power cable insulation and the influence of the interface on their properties.

The replacement of LDPE (thermoplastic polymer) with XLPE (thermosetting polymer) enhanced the thermomechanical properties of power cable insulations. Due to the crosslinking process, the thermal stability, and, hence, the long-term operation in service, was significantly improved from 70 to 90 °C [7]. XLPE is being able to withstand even short circuit conditions for a few seconds with conductor temperatures over 200 °C [8]. However, in the case of XLPE, the modulus is reduced by several orders of magnitude at operating temperatures between 90 and 100 °C [9]. Power cable insulations with low modulus are prone to irreversible mechanical damage during operation [10]. Currently, conventional XLPE is at the limit of capabilities, both in terms of purity (which influences the electrical properties, especially the electrical conductivity [11]) and thermal stability (which determines the maximum operating temperature). In order to obtain insulations with higher operating temperatures, which allow higher current densities through conductors, there are two important directions. On the one hand, PE could be replaced by other polymers such as EPR/EPM, PP and copolymers of PE and PP. In particular, the P-Laser cable represents a breakthrough in power cable systems; it is based on the high-performance thermoplastic elastomer HPTE [12]. On the other hand, nanocomposites based on PE or PP and organic or inorganic fillers can be used. Nanoparticles have a larger interfacial area compared to microsized particles, which strongly influences and determines the properties of nanocomposite materials, even at low volume concentration of such fillers (Figure 3) [13,14,15,16]. The performance of polymer-based nanocomposites is affected by particle agglomerations since nanoparticles have a strong tendency to aggregate (in particular if polar particles such as silica are dispersed in a non-polar polymer matrix such as PE). In order to avoid agglomeration and to maintain the stability of the nanoparticles within the polymer matrix, they are often surface-functionalized [14,17]. Carefully tailored interfaces of the incorporated nanoparticles enable the preparation of insulating nanocomposites with properties that exceed those of HVDC cables (from 320 to 800 kV), which are applied in industry nowadays.

Although XLPE technologies are widely adopted and expected to be continuously used in the future [10], the introduction of PE-based nanocomposites for power cables insulation is a solution taken into consideration by many cable manufacturers. For commercial use, nanocomposites have to fulfil several requirements involving improved thermomechanical and electrical properties and sustainable economic and environmental characteristics. For AC cables and their joints, the polymeric materials must exhibit, among other things, low electrical conductivity, tailor-made permittivity and low loss factor, high dielectric breakdown strength, partial discharge resistance, absence of electric and electrochemical treeing, stability at higher operating temperatures, and so forth. In the case of HVDC, the insulating materials must meet two additional essential requirements: (*i*) low variations in electrical conductivity with varying temperature and electric field intensity and (*ii*) low space charge accumulation [7,18].

The experimental studies revealed that the introduction of nanoparticles such as Al_2_O_3_, SiO_2_, TiO_2_, MgO, ZnO, carbon black, graphene, graphene oxide and so forth, lead to a significant increase in electrical resistivity (1-2 orders of magnitude [18]) and dielectric rigidity. A reduction of space charge accumulation and an increased resistance to the action of partial discharges as well as electric and water treeing were also observed. In addition to choosing the type and concentration of the particles, the properties of the nanocomposites can be conveniently adjusted by surface modification of the nanoparticles [19]. Generally, the effect of nanoparticles on reducing the electrical conductivity values and space charge accumulation is stronger if their surfaces were covered or treated by, for example, chemical modification. For example, in the case of LDPE nanocomposites with silane-coated Al_2_O_3_ nanoparticles (50 nm in diameter), the electrical conductivity dropped 50 times to that of LDPE. The greatest reduction in conductivity by two orders of magnitude was achieved by using a treatment with *n*-octyl-bearing silanes [20]. It should be noted that the introduction of nanoparticles in order to improve selected properties might adversely affect other properties of the composites. For example, the introduction of carbon black CB in LDPE causes a reduction of space charge injection and field distortion but can decrease the DC breakdown strength of the nanocomposite (the dielectric permittivity and dielectric loss remaining adjacent to LDPE without CB) [21].

The maximum value of space charge density accumulated in HVDC insulation must be relatively low in order to ensure higher reliability and long-term life performance [22]. In this case, the maximum electric field must be below the threshold for space charge accumulation [22,23]. In fact, for all the simulations on the behavior of HVDC cables, it was considered that the cables operate below the threshold for space charge accumulation (the cables are space charge free), except for the charge distribution that is the result of a temperature gradient in the insulation [22]. As mentioned above, this can be accomplished by introducing nanostructured materials based on XLPE filled with SiO_2_ or MgO nanoparticles [24]. On the other hand, the presence of moisture leads to a deterioration of the electrical properties of polymer-based nanocomposites (in particular, reduced breakdown strength and increased losses) [25,26,27]. In order to reduce the influence of humidity, a treatment of the nanoparticles can be performed, as shown in literature for MgO nanoparticles [28]. Increasing the moisture resistance of nanoparticles is due to a covalent attachment of functional silanes, which is carried out as an intermediate step after a low-temperature thermal decomposition of Mg(OH)_2_. It was found that moisture-resistant MgO nanoparticles retained their phase/structure even after extended exposure to humidity and that the addition of these nanoparticles in 1 wt % quantity into a LDPE matrix resulted in a significant increase of the electrical resistivity [28].

The use of PE based nanocomposites for commercially available high-voltage cable insulations is still in its infancy. This is due to, among other things, reduced quantities of nanodielectrics and the fact that the improvements of certain properties of these materials (electrical, thermal properties, etc.) are not always valid for other properties (mechanical properties, etc.) [29]. However, the realization of the first XLPE nanocomposite cables insulation (XLPE with MgO) should be emphasized, namely the ± 250 kV Hokkaido-Honshu LCC HVDC cable link in 2012 [30,31,32] and the ± 400 kV ones, which will be put into operation in a project connecting England and Belgium in 2019 [18]. The effect of MgO on reducing the electrical conductivity is more pronounced than that of SiO_2_ because MgO has a higher relative permittivity of ε*_r_* = 9.8 compared to that of SiO_2_ of ε*_r_* = 3.9 [18]. It should be noted that the documentation regarding the space charge behavior or mitigation on production-size transmission-class HVDC extruded cables are not yet available [24]. It should be evidenced that a combination of the data availability regarding the applications of nanocomposites and the commercial availability of ultra-clean XLPE enables the future development of HVDC cables with ultra-high voltage rating [33].

In the case of DC cables junctions with two polymer layers, it is also necessary to consider the reduction or even cancellation of superficial charge accumulated in their interfaces. For this purpose, XLPE and nanocomposite layers of EPDM with SiC can be used [34]. It should be noted, however, that the accumulated charge density increases upon the application of voltage and then decreases until cancellation [34]. The introduction of nanoparticles into PE can also lead to an improvement of the thermal conductivity of cable insulation, an important requirement for reducing their thermal ageing. For example, in the case of LDPE and polyhedral oligomeric silesquioxanes (POS) nanocomposites, an increase in thermal conductivity was achieved by approx. 8%, while the dielectric rigidity remained unmodified and the corona discharge resistance increased [35]. In addition, the introduction of boron nitride (BN) particles into LDPE resulted in an increase of the thermal conductivity of up to 1 W·m^−1^∙K^−1^ at a filler loading of 40 wt % [36].

Although a series of PE-based nanocomposites with electrical and/or thermal properties superior to the unfilled polymer have been achieved, the introduction of these new materials into the current production of power cables requires the performance of extensive testing and life modelling to investigate both the space charge trapping properties and the long-term life performance, in order to define suitable levels for the design field and reach cost-effective designs associated with the desired life and reliability levels [22].

## 3. Polymers for Power Cable Insulations

**Figure 4:**
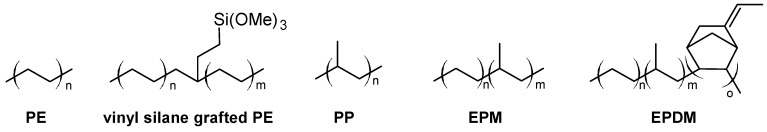
Chemical structure of polymers used as extrudable dielectric materials. PE: polyethylene; PP: polypropylene; EPM: ethylene propylene rubber; EPDM: ethylene propylene diene monomer rubber.

In the field of MV and HV cables, cable jackets and semiconducting layers, extruded polymers are commonly used. Benefiting from low raw material and processing costs together with high reliability and adequate material performance, polyethylene (PE) and, in particular, crosslinked polyethylene (XLPE) are widely applied [37]. Other polyolefins such as syndiotactic polypropylene (PP) have been reported to exhibit good insulating properties but the high cost of the material hinders widespread application [38]. Along with homopolymers, also blends of different types of polyolefins, copolymers such as ethylene propylene rubber (EPR) and ethylene propylene diene rubber (EPDM) are employed as extrudable dielectric materials. The chemical structures of selected polymers are shown in Figure 4.

### 3.1. Power Cable Insulations Based on Polyethylene (PE)

**Figure 5:**
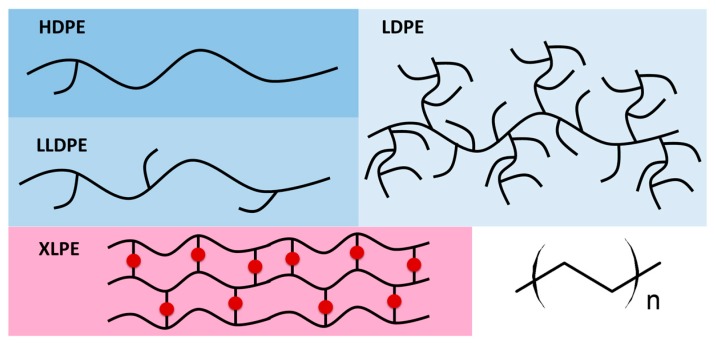
Schematic representation of the structures of varying types of PE.

Polyethylene comprises a saturated carbon-carbon backbone and is a typical thermoplast, which means that the polymer melts when heated above its melting point. The type of branching, the crystal structure and the molecular weight of the polymer chains mainly govern the material properties of PE (Figure 5). The most prominent types are low-density polyethylene (LDPE) with a considerable number of short-and long-chain branching, linear low-density PE (LLDPE) with a significant degree of short branches and high-density PE (HDPE) with a low amount of branching. LLDPE and HDPE are produced by coordination polymerization in the presence of selected catalysts (e.g., Ziegler-Natta, Philips, metallocenes), which leads to controlled branching and molecular weight of the polymer chains. In contrast, LDPE is obtained by free-radical polymerization at high pressures and high temperatures without the use of any catalyst, resulting in polymer structures with random short- and long-chain branching. Thus, the material costs of LDPE are much lower than LLDPE or HDPE, which makes it, in conjunction with the low dielectric constant, the low dielectric loss and the high breakdown strength, an ideal candidate for extrudable dielectric materials [39].

Previous work has shown that the electrical properties of LDPE such as dielectric strength and space charge formation are influenced by its crystalline structure [40,41]. LDPE is a semi-crystalline polymer typically containing 45–55% crystalline domains in the form of lamellae, which are surrounded by the amorphous bulk phase. The size of the crystalline domains can be controlled by the parameters of the extrusion process. While high cooling rates result in smaller domains and lower degrees of crystallinity, thermal annealing yields larger domains in conjunction with a higher amount of crystallinity. The annealing and cooling steps of the extruded LDPE insulation are carried out under high pressure and under inert conditions in order to reduce the formation of voids. Particularly in the production of cable insulation, higher crystallinity and smaller domains are favoured as the final product correspondingly shows smaller voids and improved ductility. Along with the crystalline regions, the amorphous bulk has also a distinctive influence on the electrical properties of LDPE. Dissado and Fothergill [42] demonstrated that charge transport mainly occurs within the amorphous regions of LDPE. Khalil [43] has shown that the initial morphology of PE can change during thermal cycling in conjunction with DC conductivity leading to a distinctive increase in conductivity.

The electrical behavior of PE is further influenced by impurities, voids and ageing (e.g., carbonyl moieties formed by oxidation of the polymer chain), which are expected to induce space charge accumulation leading to a local heating that can result in electrical breakdown of the insulation material [42].

### 3.2. Power Cable Insulations Based on Crosslinked Polyethylene (XLPE)

Aiming at an enhanced thermal and chemical resistivity in combination with improved mechanical properties (in particular at high filler loading) and ageing behavior, LDPE may be crosslinked (XLPE). Due to the crosslinking of the polymer chains, the operational temperature can be increased from 75 to 90 °C. Previous studies [39] report that XLPE is stable at 130 °C during 36 h. However, if the temperature of the conductor reaches 250 °C (e.g., during a short circuit), the XLPE-based insulation degrades within seconds [44].

**Figure 6:**
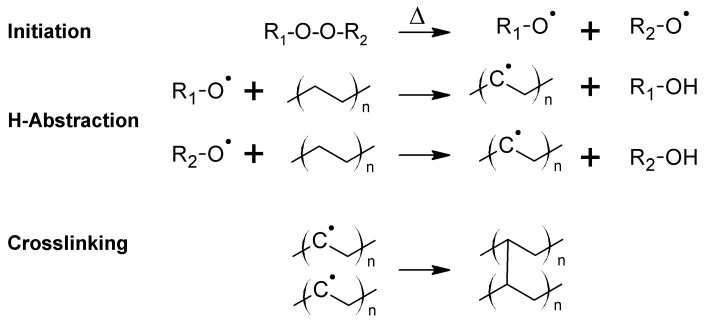
Thermal curing of PE with organic peroxides. Redrawn and adapted from reference [45].

The most common crosslinking mechanism originates from the addition of radical initiators such as organic peroxides, which undergo homolytic bond cleavage during the extrusion process and initiate radical-induced crosslinking of the polymer chains (Figure 6). Curing with dicumyl peroxide enables safe processing up to 120 °C, while the processing temperature can be increased to 150 °C using 2,5-bis-(*tert*-butylperoxy)-2,5-dimethylhexane. Thermal cleavage of dicumyl peroxide yields several by-products involving methane, acetophenone and cumyl alcohol. The curing of the extruded insulation is usually performed at high pressures in the range of 12–20 bar to avoid the formation of voids from such gaseous by-products. During the production of cables, the XLPE-based insulation is kept in a fan-forced oven at elevated temperature (70 °C) to remove the majority of these by-products (particularly methane, which is highly flammable and forms explosive gas mixtures with air) [39].

**Figure 7:**
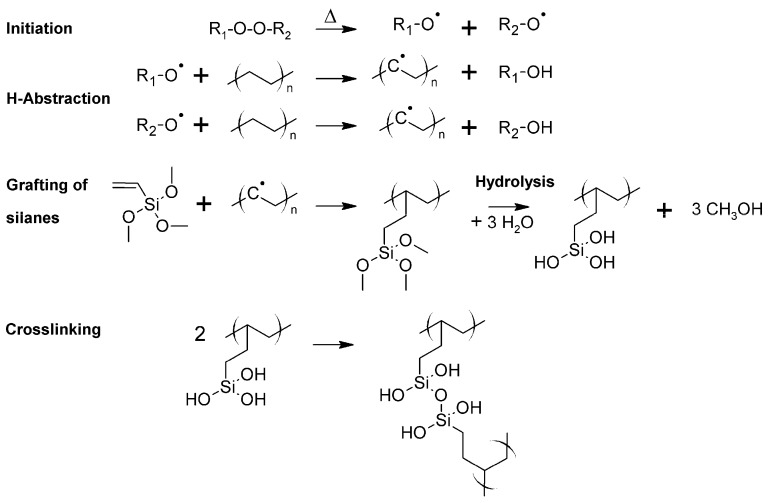
Thermal curing of PE with siloxanes. Redrawn and adapted from reference [48].

Another well-established approach involves the crosslinking of a chemically modified PE in the presence of a catalyst and moisture after the extrusion process (Figure 7). The chemical functionalization of the PE is carried out by grafting vinyl silanes onto the polymer chain during the extrusion process. Small amounts of an organic peroxide are added to facilitate the grafting process [46]. Modified PE grades, which are produced by copolymerizing ethylene and 3-vinyltrimethoxysilane, are commercially available [47]. After the extrusion of the modified PE, the cables are stored in a water bath at high temperatures or in a steam chamber to induce the crosslinking reaction. The crosslinking reaction, which involves a hydrolysis reaction followed by a condensation of the generated silanol groups, is catalyzed by dibutyltin dilaurate.

**Figure 8:**
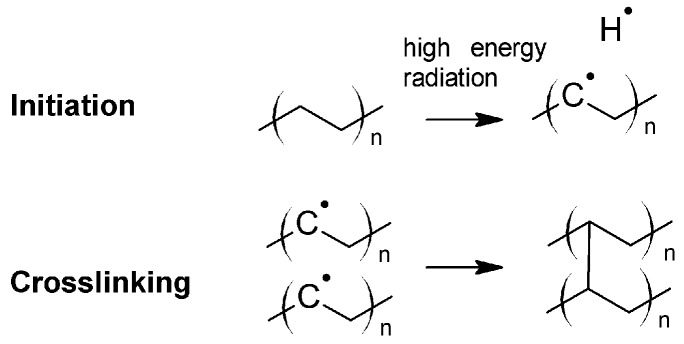
Crosslinking of PE with high energy radiation. Redrawn and adapted from reference [52].

In addition, crosslinking of LDPE is also obtained under high energy radiation such as electron beam and gamma radiation generated from a Co^60^ source [49,50,51]. The crosslinking is based on a free-radical mechanism involving the extraction of a hydrogen atom from the polymer chain by the accelerated electrons or by the electromagnetic wave (Figure 8). Polymer radicals are formed, which recombine under the formation of a covalently bound crosslink site. In order to increase the degree of crosslinking, sensitizers such as acrylates may be added to the polymer. The radiation induced crosslinking is carried out after the extrusion of the insulating layer at ambient conditions. Aiming to avoid a rapid temperature increase during crosslinking, the extruded cables are passed through the electron beam of electron radiation over several cycles until the targeted exposure dose has been reached. In general, crosslinking of PE with high energy radiation has the disadvantage of high processing costs, as special radiation sources at high investment costs are required.

### 3.3. Power Cable Insulations Based on Other Classes of Polymers

Along with XLPE, ethylene-propylene rubbers have been the most popular dielectric materials in extruded cables over the last decades [39]. They can be divided into two main classes: (*i*) ethylene-propylene rubber (EPM or also EPR) as a copolymer of ethylene and propylene and (*ii*) EPDM as terpolymer, which consists of ethylene, propylene and diene components such as dicyclopentadiene, ethylidene norbornene, and/or vinyl norbornene.

EPR is a fully saturated and nonpolar polymer with high temperature stability and high resistivity towards oxidation and polar solvents. EPR congeners with a low ethylene content are amorphous and easy to process but typically have inferior mechanical properties. In contrast, EPR types with a high ethylene content are semi-crystalline and have improved mechanical properties. Similar to LDPE, EPR may be crosslinked with organic peroxides [53,54].

EPDM has a fully saturated polymer backbone but additionally comprises unsaturated carbon-carbon bonds in the side-chains, which change the reactivity of the polymer in crosslinking reactions. In addition to curing with peroxides, EPDM can be also cured by sulphur vulcanization involving the unsaturated carbon-carbon bonds. While the electrical properties of sulphur- and peroxy-crosslinked EPDM are comparable, it was demonstrated that sulphur-cured EPDM insulations show poor performance after long-term immersion in hot water. In extruded EPM or EPDM insulations, the polymer content is typically in the range of 50%, as a high amount of inorganic fillers (e.g., clay, talc, silica, and alumina) is added to yield smooth surfaces and sufficient mechanical strength of the final insulation.

## 4. Nanocomposites for Power Cable Insulations

**Figure 9:**
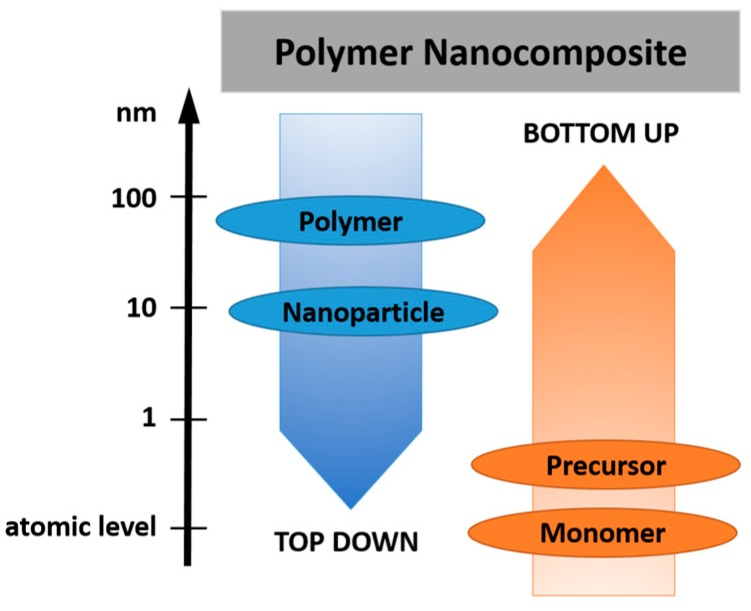
Differences between ‘bottom up’ and ‘top down’ processes used in the preparation of polymer nanocomposites.

In order to tune the electrical and mechanical properties of extruded polymers, the addition of selected nanosized inorganic and organic fillers has gained increased attraction. These so-called nanocomposites benefit from (*i*) the low weight, (*ii*) the easy processing and (*iii*) shaping of the polymer matrix as well as (*iv*) the salient properties of the incorporated nanoparticles, which are substantially different to their micrometer-scaled counterparts. At a given volume, nanosized fillers have a distinctively larger surface area than microsized ones. As the chemical and physical properties of composites are strongly influenced by the interactions between the filler and the polymer matrix, nanofillers yield different properties than macroscopic particles of the same chemical and morphological composition. This effect is also exploited in the cable industry and numerous studies have been reported on the production, characterization and performance of nanocomposites as dielectrics in cables [14,33,55,56]. The following section gives a short summary describing the types of nanofiller and the preparation of nanocomposites following ‘bottom up’ and ‘top down’ processes (Figure 9).

### 4.1. Fillers Used in Nanocomposites

**Figure 10:**
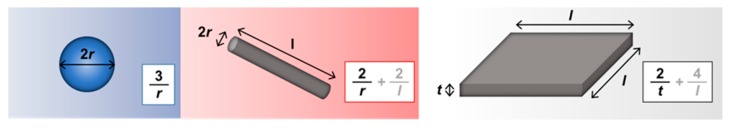
Three main types of nanofillers (from left to right: spherical particles, fibers and platelets), classified by their geometry and area-to-volume ratio. Redrawn and adapted from reference [57].

It is well known that the high aspect-ratio of nanofillers mainly contributes to their reinforcing efficiency. Depending on the geometry of the particles, three main types of fillers are distinguished (Figure 10): (*i*) (spherical) particles, (*ii*) fibers and (*iii*) platelets [57,58]. In terms of fibers and platelets, the (surficial) area-to-volume ratio is mainly governed by the first term (2/r and 2/t) of the equation, while in nanomaterials, the influence of the second term is negligible. Thus, a change of the particle geometry from the micro- to nanometre size changes the area-to-volume ratio by three orders of magnitude.

Along with the geometry, nanofillers may also be classified either by their chemical and morphological structure or by their origin (natural versus synthetic and organic versus inorganic) as shown in Table 1.

The properties of nanocomposites are not only influenced by their geometry and type but also by the dispersion of the filler in the polymer matrix. Nanofillers tend to agglomerate during the preparation of nanocomposites, which compromises the electrical, mechanical and optical properties of the final material [59]. In order to improve the dispersion of the particles in the polymer matrix and to ensure an enhanced bonding between the particles and the polymer matrix, surface modification of the particles is often carried out [60].

### 4.2. Methods for the Preparation of Nanocomposites

**Figure 11:**
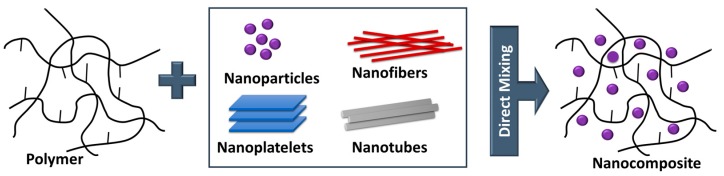
Preparation of nanocomposites by direct mixing.

Over the last years, four main routes have been established for the successful incorporation of inorganic nanofillers into a polymer matrix: (*i*) direct mixing of polymer and filler, (*ii*) intercalation based on the exfoliation of, for example, layered silicates, (*iii*) sol-gel processes and (*vi*) in-situ formation of nanofillers in the polymer matrix [59,62,63]. The simplest route involves a direct mixing of the nanoparticles in the polymer, above the glass-transition temperature *T*_g_ or the melting point *T*_m_ of the polymer (melt-compounding method). Alternatively, the direct mixing can be also carried in a polymer solution (solution-mixing method). After evaporation of the solvent, the fillers are well distributed in the polymer matrix. Direct mixing is a typical top-down process, which means that energy is used (i.e., mixing energy) to transform a bulk material in smaller fragments until a nanocomposite is obtained (Figure 11) [64,65].

**Figure 12:**
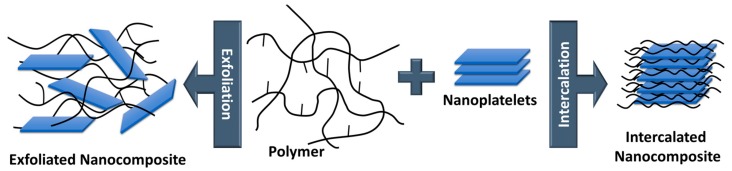
Preparation of nanocomposites by exfoliation and intercalation. Redrawn and adapted from reference [67].

Another top-down process is the intercalation involving the exfoliation of layered silicates (Figure 12). Three different methods are typically pursued: (*i*) direct intercalation of polymer chains from solution, (*ii*) polymer melt intercalation and (*iii*) intercalation of monomers followed by in-situ polymerization [66].

Regarding the direct intercalation of polymer chains from solution, the layered fillers (typically nanoclays) are dispersed into a solvent in which the polymer is soluble [68,69]. The solvent migrates through the layers of the filler to start the exfoliation. After evaporation of the solvent, single clay platelets are well dispersed in the polymer matrix. With respect to melt intercalation, the layered fillers are directly mixed with the polymer melt. Due to shear forces, the exfoliation of the platelets starts and, if the surface polarities of filler and polymer are similar, the polymer chains migrate into the interlayer space. In terms of intercalation of monomers followed by in-situ polymerization, monomers and selected initiators are employed [69]. The monomers intercalate into the layered filler and increase the distance between the layers. Subsequent polymerization of the monomers leads to an exfoliation of the filler and polymer-based nanocomposites are yielded.

**Figure 13:**
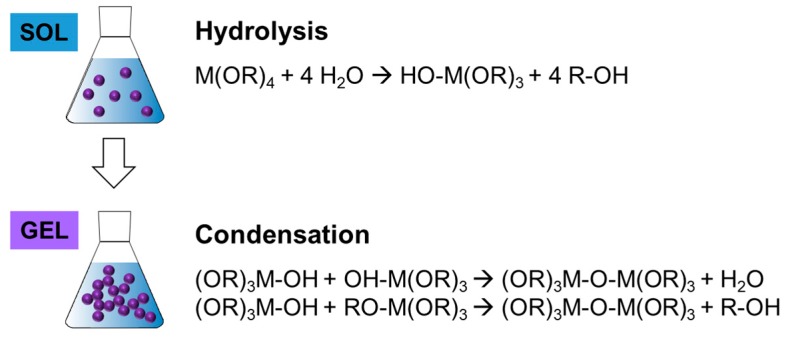
Preparation of nanocomposites by sol-gel processes. Redrawn and adapted from reference [71].

The sol-gel process is a prominent example of a bottom-up approach, which involves the building of the targeted material by the assembly of building units (e.g., atom-by-atom or cluster by cluster) [68,70]. The sol-gel process relies on two subsequent reactions steps (Figure 13). In the first step, metal oxides are obtained from the hydrolysis of organic metal alkoxides or esters yielding a colloidal suspension of solid particles in a liquid phase (sol). In a second step, the hydrolyzed intermediates start to condensate forming an interconnected network (gel) between the particles.

**Figure 14:**
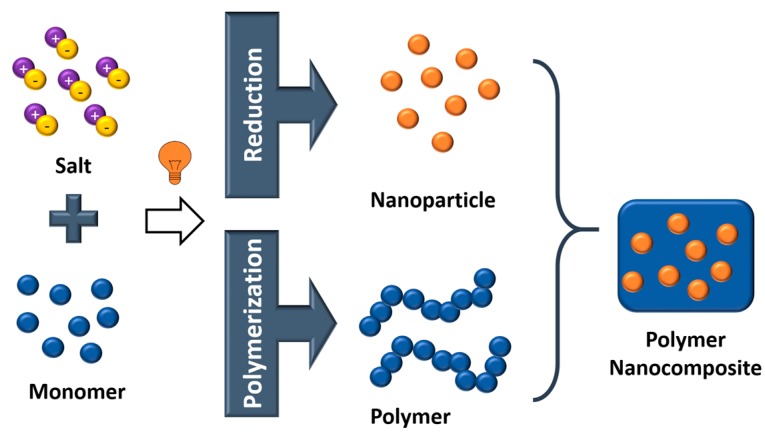
Preparation of nanocomposites by UV-induced in-situ generation of nanoparticles and in-situ polymerization of a photoreactive monomer.

The fourth method involves the in-situ generation of nanoparticles from metal ions by redox reactions, which can be stimuli-triggered by a change of the pH value or by UV light (Figure 14). The in-situ generation of the nanoparticles is usually carried out in conjunction with an in-situ polymerization using colloidal sols with metal ions and monomers. This approach is typically employed to obtain nanocomposites from thermosetting resins such as epoxides or photocurable resins such as acrylates [72,73,74].

### 4.3. Surface Modification of Fillers to be Used in Nanocomposites

The homogeneous dispersion of nanosized fillers within the polymer matrix has major influence on the final properties of the nanocomposites [59]. In particular, the surface modification of inorganic particles has become a popular route to avoid agglomeration and cluster formation of nanofillers, since the attachment of functional groups on the particles’ surfaces enables the controlled change of polarity and reactivity of the particles’ surfaces. A typical example is the surface modification of carbonates or silicates with hydrophobic fatty acids to improve the dispersibility in non-polar polymer matrices such as polyolefins [75]. Besides the dispersibility, the particle-polymer interfaces can be tailored by incorporating functional groups on the fillers’ surfaces. As the particle-polymer interface has a crucial influence on the performance of the corresponding nanocomposites, surface modification techniques have gained increased attention for tuning the mechanical and electrical performance of polymer nanocomposites [76,77,78,79]. Pallon et al. [80] applied functional silsesquioxane coatings on MgO nanoparticles and incorporated the functional filler in LDPE. They demonstrated that the modified particles were homogenously distributed within the polymer matrix and, by adding only 3 wt % of the surface-treated particles, the volume conductivity was decreased by two orders of magnitude. For the modification of inorganic particles, different strategies are pursued involving (*i*) chemical treatment, (*ii*) grafting reactions and other methods such as (*iii*) adsorption of polymeric dispersants [81].

**Figure 15:**
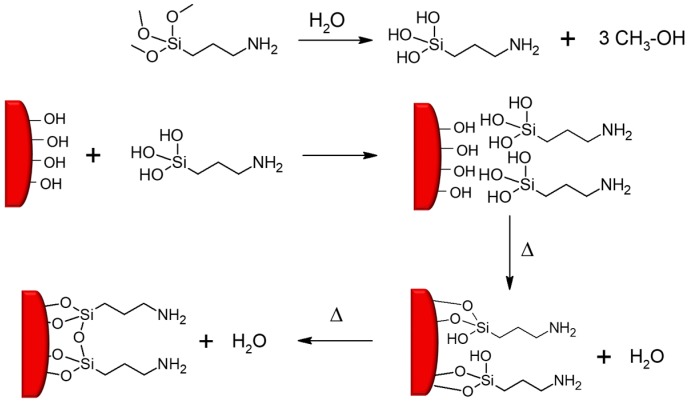
Covalent attachment of functional silanes on nanosized silica. Redrawn and adapted from reference [86].

The typical chemical surface modification reaction proceeds in one step using bifunctional organic compounds with one group that reacts with the nanoparticles’ surfaces and a second group, which represents the functionality of the organic shell. One well-established approach is the so-called silanization, in which functional trialkoxysilanes such as 3-aminopropyl triethoxysilane are covalently attached to surficial hydroxyl groups of inorganic particles (e.g., SiO_2_, TiO_2_, Al_2_O_3_, ZnO, Fe_3_O_4_) by condensation reactions (Figure 15) [82,83]. In terms of carbon-based nanofillers such as carbon black, fullerenes, carbon nanotubes or graphene, Diels-Alder reactions can be employed to change the surface characteristics of the particles [84]. If the functional groups of the organic compound are not compatible with the synthetic process, a step-wise procedure may be carried out for the modification of inorganic particles [85].

**Figure 16:**
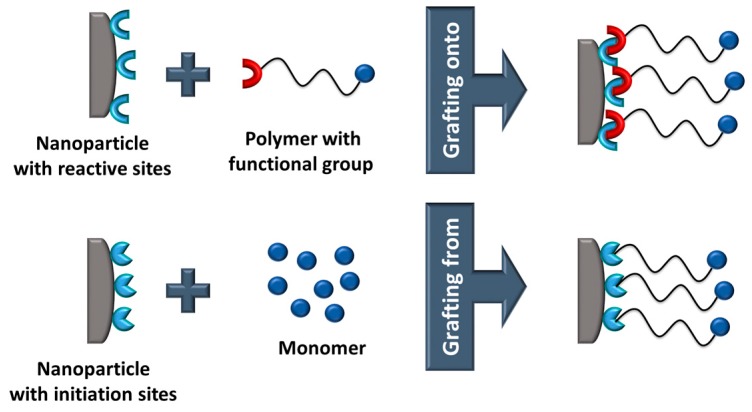
Modification of nanoparticles via (*i*) ‘grafting onto’ and (*ii*) ‘grafting from’ reactions.

Grafting reactions represent another route to modify the surface of inorganic particles (Figure 16) [86]. The grafting mechanism involves either (*i*) direct coupling of a polymer chain onto the particle surface (‘grafting onto’ reactions) or (*ii*) immobilization of a monomer or an initiator on the particle surface, which is followed by a polymerization of reactive monomers (’grafting from’ reactions).

A convenient method for surface modification of inorganic nanoparticles involves the physical adsorption of polymeric dispersants, which is typically used to enhance the dispersion stability of nanoparticles in solvents [87,88]. The improved dispersion properties mainly rely on the steric repulsive forces between the adsorbed polymer chains and the related increase in surface charges.

## 5. Electrical Conductivity of Nanocomposites

### 5.1. General Aspects of Electrical Conduction

Electrical conductivity is an intrinsic property that quantifies the ability of materials to conduct electric current [89,90] and can be classified in three major categories: (*i*) *intrinsic conductivity:* charge carriers are generated based on the chemical structure of the material; (*ii*) *extrinsic conductivity:* charge carriers are generated by impurities, which can be introduced during the fabrication process or by dopants through specific methods; (*iii*) *injection-controlled conductivity:* charge carriers are injected into the material through the interface between the metallic electrodes and the non-metal material.

**Figure 17:**
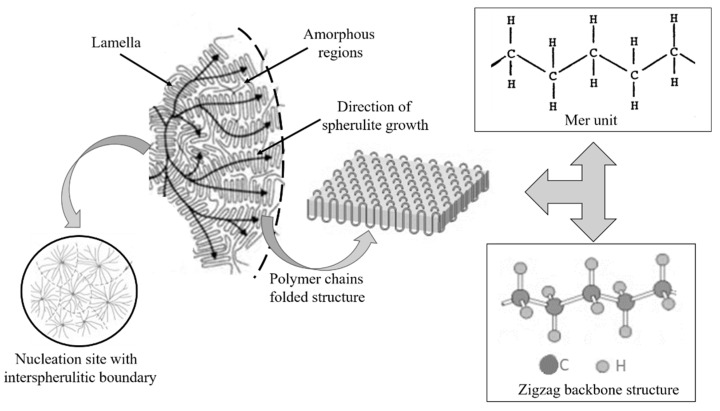
The morphological structure of PE. Redrawn and adapted from reference [92].

Regarding insulators, the charge carriers’ origins for intrinsic and extrinsic conductivities are not well distinguished; in polymeric insulators, the situation is even less well characterized and understood. Some polymeric materials such as PE can be considered as natural nanodielectric material with contrasting conductive crystallites and resistive amorphous regions of nanometric dimensions [91], as it was described in Section 3 (Figure 17).

In the crystalline phase of PE, intrinsic conduction is improbable due to the large band gap of approx. 8 eV and the corresponding separation of electrons and holes [91]. Excluding any material defects caused by impurities, conduction in PE can only originate from extrinsic charges introduced by the injection process. Hole conduction commonly appears in PE, which suggests that hole injection at the anode occurs more easily compared to the electron injection at the cathode [91]. However, the crystalline regions in PE are surrounded by amorphous regions and the transfer of electrons and holes between them is likely to be hindered. Holes enclosed to the valence band will move along the crystallites’ chain paths and will become trapped in the interphase between them and the amorphous regions. However, the transition of holes through this interphase will occur by tunnelling due to a super-exchange between donor and acceptor hole traps [91]. Amorphous regions in PE are considered to have high concentrations of traps introduced by impurities and additives, which may be polarized and maybe even move through them. Hence, extrinsic conduction (ionic transport) is more likely to occur in the amorphous phase [93].

Lewis et al. [91] concluded that the incorporation of oxide nanoparticles in PE causes a strong decrease of the local hole inter-lamella transition rate. They assumed that the tunnelling of holes between lamellae through the amorphous phase in the neighborhood of a particle was strongly influenced by the embedded particle and its surrounding interface. The magnitude of affected transitions, which would lead to macroscopic decrease of mobility and conductivity, would depend on the concentration of nanoparticles embedded within the polymer matrix. In agreement with this conclusion, several experimental results regarding the electrical conductivity of thermoplastic nanocomposites based on polyethylene used as power cables insulation will be presented in the following sections.

### 5.2. Conductivity of Nanocomposites Based on Polyethylene (PE)

The incorporation of various oxidic nanoparticles (e.g., Al_2_O_3_, SiO_2_, TiO_2_, MgO, ZnO, etc.) in PE delivers advantages such as a significant reduction in electrical conductivity for a certain range of nanoparticle concentrations (usually between 0 and 5 wt %). This reduction reflects the ability of the polymer matrix to incorporate the nanoparticles within the inter-lamellae spaces. Above this limit, the excess of nanoparticles is likely to be incorporated in inter-spherulite regions, which does not directly influence the hole conduction from inter-lamellae crystallites [91].

**Figure 18:**
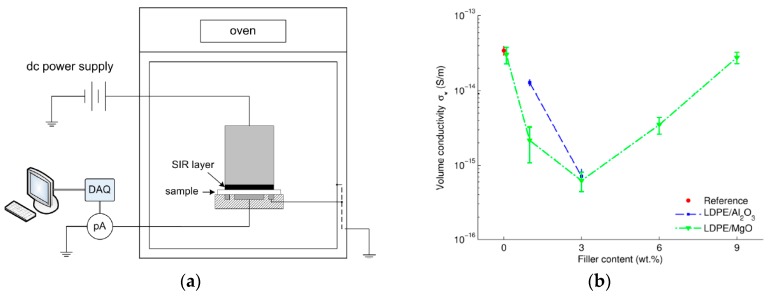
(**a**) Schematic representation of the test setup for DC conductivity measurements; (**b**) Variations of the DC conductivity with the filler concentration of the analysed nanocomposites. Reprinted from reference [94].

Hoang et al. [94] analyzed the bulk conductivity of LDPE and its nanocomposites with uncoated magnesia (MgO) and alumina (Al_2_O_3_). The investigations were performed on thin films prepared by thermal extrusion at 150 °C from a dried powder mixture of LDPE, nanoparticles and antioxidant (0.02 wt % of Irganox 1076). Two types of nanocomposites based on LDPE filled with 1 and 3 wt % of Al_2_O_3_, as well as five types of LDPE filled with 0.1, 1, 3, 6 and 9 wt % of MgO were prepared. The DC conductivity measurements were carried out at an applied electric field of approximately 30 KV·mm^−1^, for 11 h. The measurements were conducted at isothermal conditions (room temperature, 40 and 60 °C) by placing the electrode system with the sample inside to a grounded oven (Figure 18a). DC conductivity values were computed from the charging current data registered during 11 h of measurements (Figure 18b). The results on LDPE samples agreed with the data reported in literature [95]. In the case of LDPE/Al_2_O_3_ samples, the reduction in DC conductivity was proportional with the filler concentration increasing up to 3 wt %. For nanocomposites based on LDPE and MgO, a threshold-like behavior was observed around a nanofiller content of 3 wt % (Figure 18b). If the filler concentration exceeded 3 wt %, further loading with nanoparticles caused a negative effect. This change in the electrical conductivity behavior can be explained by the agglomeration of nanoparticles in the polymer matrix during the manufacturing of the samples [94]. This effect was also reported by Ishimoto et al. [96], Masuda and Murakami et al. [97,98], who described a decrease of electrical conductivity of more than one order of magnitude and a threshold of nanofillers content of approximately 2 wt %.

**Figure 19:**
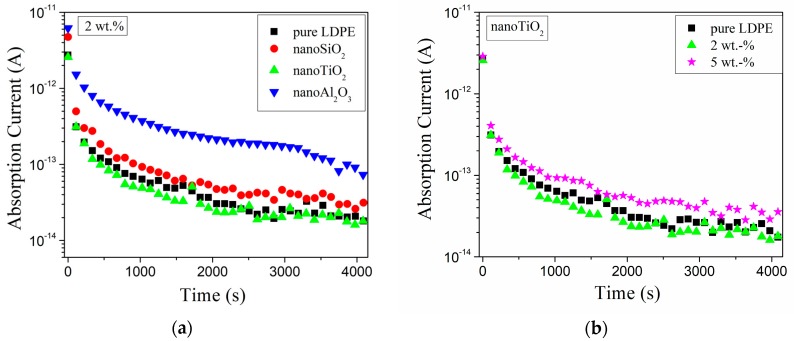
Variations of absorption currents on time for: (**a**) LDPE nanocomposites with 2 wt % of different types of inorganic nanofillers and (**b**) LDPE nanocomposites with 2 and 5 wt % of nano-TiO_2_. Reprinted, with permission by the author, from reference [99].

Pleşa analyzed the absorption currents and computed the relative DC volume resistivity of nanocomposites based on LDPE with different types of inorganic nanofillers (SiO_2_, TiO_2_, Al_2_O_3_) and various concentrations (2, 5 and 10 wt %) [99]. For better compatibility and dispersion of nanoparticles within the polymer, the surface of the nanofillers was treated with maleic anhydride. All measurements were performed at ambient temperature of 27 °C and relative humidity of approx. 50% (Figure 19). Noteworthy, the absorption currents decrease over time as a result of the reduction of the charge carrier’s concentration corresponding to bound charges (electric dipoles) and space charge. On the other hand, according to the nanocomposite models presented in literature [100,101,102], the introduction of nanoparticles into the polymer facilitates an increase in the concentration of the electric dipoles (i.e., especially inside the nanoparticles and/or inside the polymer-nanoparticle interfaces) and also an accumulation of space charge due to the huge area of polymer-nanoparticles interfaces. In this case, polarization and space charge components of absorption currents increase with enhanced concentration of nanofillers, except for LDPE with 2 wt % of nano-TiO_2_ that showed lower values compared with all the other types of analyzed materials (Figure 19a,b).

**Figure 20:**
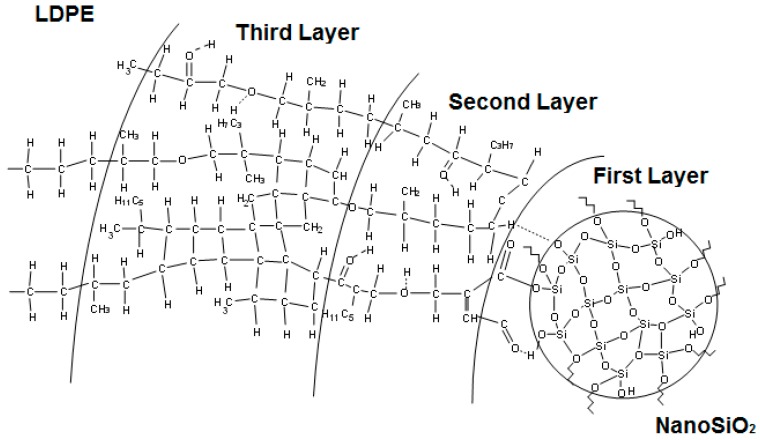
Schematic representation of an interfacial LDPE-nano-SiO_2_ structure. Reprinted, with permission by the author, from reference [99].

In order to explain the variations of the currents, models of the nanocomposites’ structure were used. Starting from the structural models proposed by Tanaka et al. [100] as well as Lewis [103], a new model for nanocomposites based on LDPE with spherical inorganic fillers was developed (Figure 20) [99]. It was considered that the interface was formed by three distinct regions: a bonded first layer, a bound second layer and a loose third layer, with an electric double layer overlapping these three layers. The polymer is in an intimate contact with the nanoparticle surface within the first layer, while the second layer represents an interfacial region. The third layer interacts superficially with the second layer and the properties of this region are supposed to be similar to the polymer matrix.

**Figure 21:**
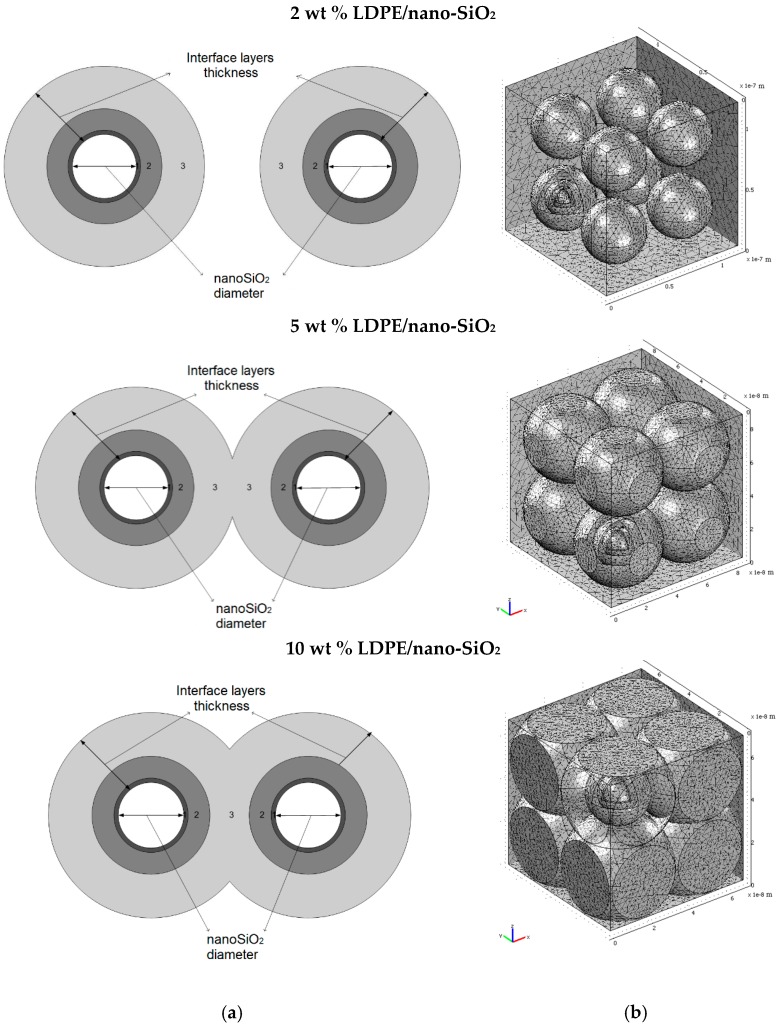
(**a**) Two neighbor nanoparticles within LDPE composites for different filler contents; (**b**) 3D electrostatic model of nanocomposites based on LDPE with nano-SiO_2_ in different concentrations. Reprinted, with permission by the author, from reference [99].

In case of LDPE nanocomposites with 2 wt % of nano-TiO_2_, a decrease of absorption currents compared to unfilled LDPE values was registered (Figure 19a). This behavior could be explained by the presence of crosslinked polymer in the third layer of the polymer-nanoparticle interface, which reduces the mobility of space charges through the material structure and may lead to a decrease of the space charge component of the absorption current values. Between the end groups of the polyethylene chains from the second layer of the interface and the surface of the functionalized inorganic nanoparticles, hydrogen bridges are formed that reduce the mobility of polymer chains and decrease the polarization component of the absorption current. Tanaka et al. [100] stated that within the intermolecular regions of the interface layers, the introduced traps were distributed as follows: deep traps in the first and second layer and shallow traps in the third layer. By increasing the filler concentration over a certain limit (i.e., >2 wt % in the case of LDPE/nano-TiO_2_) inside the polymer matrix, the overlapping zone of filler-polymer interfaces increases, too (i.e., similar with the model presented in Figure 20 and Figure 21a,b). By applying an electric field, the electrical conduction can occur either by charge carriers jumping on the shallow traps from the interfaces, overlapping the neighbor nanoparticles or by tunnelling [99], when the distance between the traps is below the minimum threshold tunnelling, leading, in overall consequence, to an increase in space charge components of the absorption currents (Figure 19b).

The volume resistivity can be calculated according to Equation (1):

(1) $$\rho_{v}\;=\;\frac{S}{d}\cdot \frac{U}{I}$$

in which $\rho_{v}$ is the volume resistivity (Ω·m), *S* the electrode surface (m^2^), *U* the applied voltage (V), *I* the average current after 4000 s starting from the voltage application (A) and *d* the sample thickness (m). It was observed that the resistivity values depend on the type and concentration of nanoparticles and decrease compared to unfilled LDPE (Table 2). With respect to LDPE with 2 wt % of nano-TiO_2_, the DC relative volume resistivity increases by 0.07 percent points compared to unfilled LDPE.

**Figure 22:**
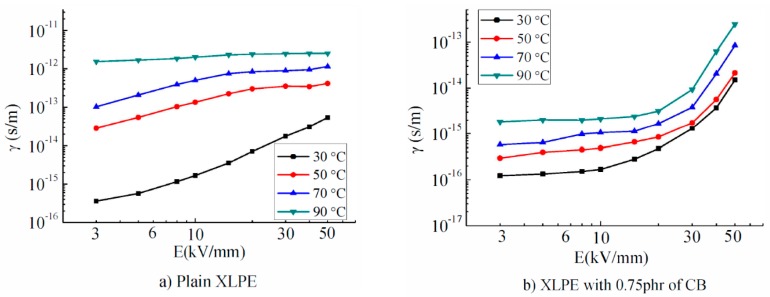

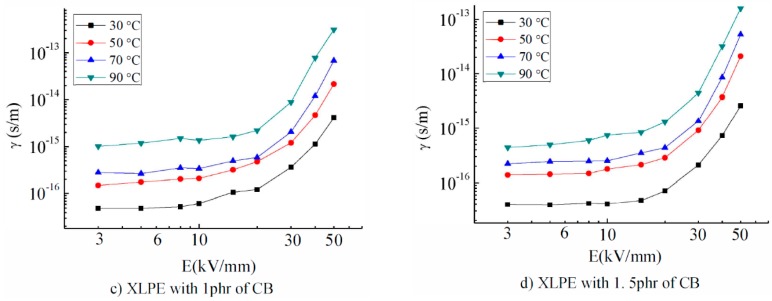
Electrical conductivity [γ(S/m)] variations of XLPE and CB/XLPE nanocomposites at different temperatures. Reprinted, with permission by IEEE, from reference [104].

In order to increase the DC electrical properties of XLPE insulation materials, Yan et al. prepared nanocomposites based on XLPE with different loadings of carbon black (CB) by the melt-blending method [104]. The space charge distribution was analyzed together with the dependence of DC electrical conductivity γ on the electric field *E* at several temperatures *T* (Figure 22). For low electric fields, a non-linear dependence of γ and *E* exists, concomitant with a significant increase in conductivity with increasing *T*. For *E* < 20 kV·mm^−1^, the electrical conductivity of XLPE/CB is almost constant; however, if the temperature increases over 30 °C, γ(*E*) decreases. This study shows that the presence of CB in XLPE can inhibit the space charge accumulation and the electric field distortion, improving the DC conductivity of XLPE/CB nanocomposites.

The correlation of the electrical conductivity with the content of nano-SiO_2_ particles in XLPE was analyzed by the CIGRE Working Group D1.24 [105]. It was concluded that the addition of fumed nano-SiO_2_ to XLPE reduced the DC conductivity of the nanocomposites. This behavior supports the mechanism describing nanofillers as charge carrier traps; it may be argued that functionalization may strengthen their function. Wang et al. [106] analyzed the electrical resistivity of nanocomposites based on XLPE containing different concentration of nano-TiO_2_ (i.e., 1, 3 and 5 wt %). It was found that their volume resistivity was higher compared to pure XLPE and increased with the filler concentration. This behavior was referred to the large number of traps introduced in nanocomposites by the fillers, which capture the charge carriers and prevent the movement of carriers within them. Murata et al. [107] investigated the volume resistivity and space charge distribution in nanocomposites obtained by mixing XLPE with MgO nanofillers. Four types of materials were analyzed: conventional XLPE insulation for AC cables (XLPE^C^), nanocomposites obtained by mixing XLPE^C^ with nano-MgO (XLPE^C^/MgO), a special type of XLPE with a lower degree of crosslinking by-products compared to XLPE^C^ (XLPE^S^) and nanocomposites obtained by mixing XLPE^S^ with nano-MgO (XLPE^S^/MgO). It was concluded that the presence of by-products (that decrease the volume resistivity) and of nano-MgO (that increases the volume resistivity) acted synergistically within XLPE-based nanocomposites, converting them into excellent materials for DC power cables insulation.

According to the experimental studies presented herein above, two types of PE-based nanocomposites are recommended for power cables, those with low conductivity for insulations (including inorganic particles) and those with increased conductivity for semiconductor layers (including carbon black fillers).

## 6. Permittivity and Loss Factor of Nanocomposites

### 6.1. General Aspects of the Complex Dielectric Permittivity

Synthetic polymers are complex molecules comprising a huge number of covalently bound atoms within the macromolecular chains, yielding numerous possible conformations of the individual macromolecular chains in space and time [108]. Due to this large number of conformations, most of the polymers have properties depending on the chain flexibility, the end-to-end vector of the chain, the mean-square dipole moment per molecule and so forth. Correspondingly, their behavior in solution and/or in solid state is analogously complex. Besides systems composed of linear macromolecules, a comprehensive variety of the molecular architectures additionally exists (e.g., branched and hyperbranched polymers, cyclic macromolecules and oligomers, polymers with star-shaped and comb-like structures, copolymers, dendrimers, etc.), which can cause new morphologies in the dense state of these molecules (i.e., phase- or microphase-separated structures).

Since Debye published the theory of dipolar relaxation in 1929 [109], the study of the interaction between electromagnetic radiation and (soft) matter, which is of crucial importance in fundamental and applied science, has been applied to dielectric spectroscopy. This experimental technique is very useful for studying the conformation, the structure and the dynamics of polymers (i.e., dipolar processes) and to evaluate the behavior of polymeric systems over a large range of frequencies and temperatures. Dipolar processes include *very low frequency processes* (i.e., electric charge transport), *Maxwell-Wagner polarization processes* (i.e., charge trapping at interfaces) and *relaxation processes* due to the motion of dipoles groups (i.e., dipole reorientation) [93].

For dielectric spectroscopy studies, one key parameter is the relative complex dielectric permittivity $\varepsilon^{*}$ (Equation (2)) [108]:

(2) $$\varepsilon^{*}\left(f\right)\;=\;\varepsilon^{\prime }\left(f\right)-i\varepsilon^{\prime \prime }\left(f\right)$$

in which $\varepsilon^{\prime }$ represents the real part of complex relative permittivity and $\varepsilon^{\prime \prime }$ the imaginary part of the relative permittivity, the so-called loss part.

The complex relative permittivity is defined as a factor between an outer alternating electric field $\vec{E}\left(f\right)$ and the induced electric polarization P of the medium (Equation (3)):

(3) $$\vec{P}\left(f\right)=\chi^{*}\varepsilon_{0}\vec{E}\left(f\right)$$

in which χ* = ε*(*f*) − 1 is the complex electric susceptibility of the material and ε_0_ = 8.85·10^−12^ F·m^−1^ is the vacuum permittivity.

The complex relative permittivity is a material property depending on frequency, temperature, pressure and structure of the material. According to statistical mechanics, both quantities, namely ε′ and ε″, have a physical interpretation: $\varepsilon^{\prime }$ is related to the energy stored reversibly within the material and $\varepsilon^{\prime \prime }$ is related to the energy dissipated within the material. In dielectric relaxation spectroscopy, the dissipation factor $\tan\delta =\varepsilon^{\prime \prime }/\varepsilon^{\prime }$, in which δ is the phase angle between the applied voltage and the resulting electric current, is one parameter for the discussion of the electrical performance of polymer-based materials [108].

The addition of nanoparticles into polymers considerably changes the dielectric behavior due to the formation of interaction zones within the nanocomposites [93]. Correspondingly, there has been a steadily growing interest over the last two decades to analyse these materials, which are referred to as nanodielectrics in this context. In the following section, selected results are presented regarding the permittivity and loss factor of thermoplastic polymer nanocomposites used as power cables insulation (LDPE, XLPE, etc.).

### 6.2. Permittivity and Loss Factor of Nanocomposites Based on Polyethylene (PE)

A huge number of studies have reported improved dielectric properties of thermoplastic nanocomposites with inorganic nanoparticles compared to the unfilled polymer [69,110,111,112,113,114,115,116], which render these nanocomposite materials as interesting candidates for high-voltage applications. The dielectric behavior of thermoplastic nanocomposites systems based on LDPE containing different types and contents of nanofillers have been analysed and presented in the literature.

**Figure 23:**
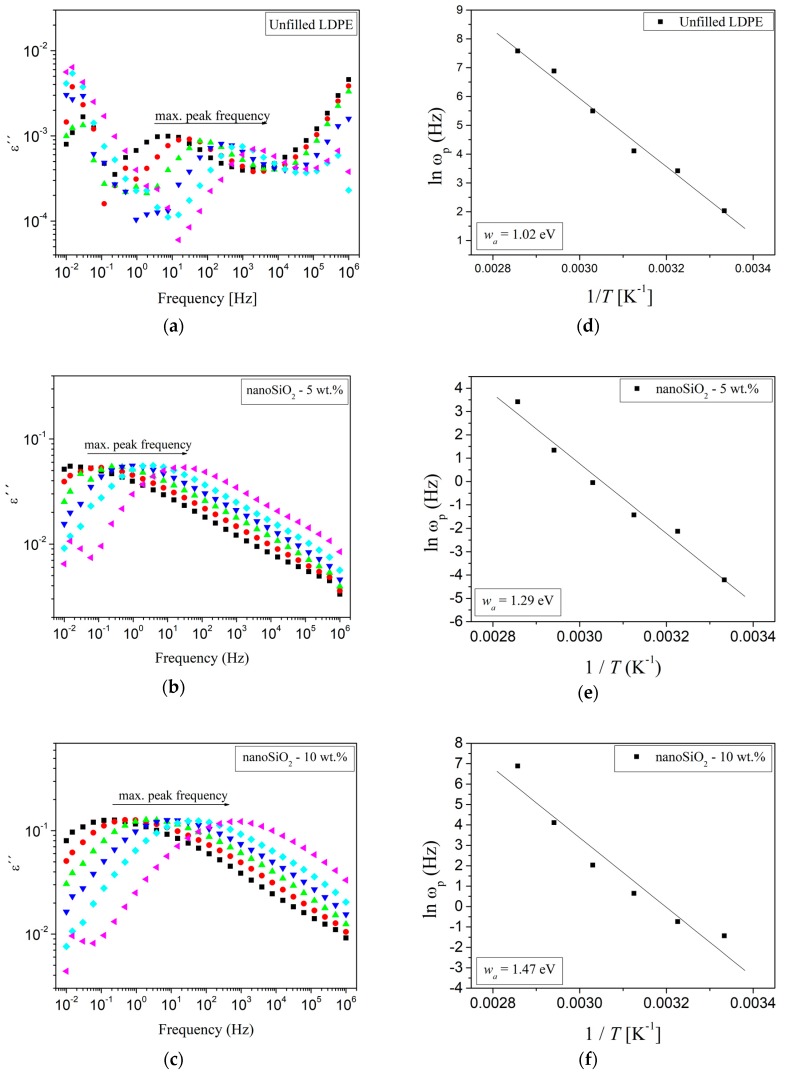
(**a**)–(**c**): Frequency variation of the imaginary part of the relative complex permittivity (ε″) of unfilled LDPE and filled with different concentrations of nano-SiO_2_ at (■) 300 K, (●) 310 K, (▲) 320 K, (▼) 330 K, (♦) 340 K, (►) 350 K. (**d**)–(**f**): Dependence of the temperature function (ln ω_p_(*T*)) for unfilled LDPE and filled with different contents of nano-SiO_2_. Reprinted, with permission by the author, from reference [99].

Pleşa et al. analyzed the behavior of unfilled LDPE and silica-filled LDPE samples [99]. Nanocomposites with different contents of nanofillers ranging from 0 to 10 wt % were prepared and characterized at different temperatures in the range from 300 to 350 K and frequencies from 10^−2^ to 10^6^ Hz (Figure 23). The activation energy *w*_a_ was estimated, and correlations with the polarization mechanisms were found. The imaginary part of the complex permittivity ε″ was determined as the product of the real part of complex permittivity ε′ and the loss factor tanδ. It was assumed that the conduction losses were substantially lower than the polarization losses by deformation and orientation. In the case of unfilled LDPE (Figure 23a), peaks in the frequency range from 10^1^ to 10^3^ Hz were shifted to higher frequencies upon temperature increases. With an increasing concentration of SiO_2_ nanoparticles in the polymer matrix (Figure 23b,c), a shift of the ε″ peaks towards lower frequencies as well as their intensification compared to the base polymer were observed. For example, in case of the nanocomposites containing 5 wt % of nanoSiO_2_ (Figure 23b), the peaks were in the range of 10^−1^ to 10^1^ Hz. When the concentration of the nanoparticles was increased, the frequency domain into which the peaks shifted increased to 10^3^ Hz in the case of nanocomposites with 10 wt % of nano-SiO_2_ (Figure 23c). Hence, the peaks occur at low frequencies in the range of 10^−1^ to 10^3^ Hz, a frequency range in which polarization losses by deformation are neglectable; it was concluded that the losses were caused exclusively by polarization losses from (re-)orientation.

According to the Debye model for dipolar relaxation of ideal dielectrics, the imaginary part of the relative complex permittivity ε″ as a function of temperature *T* is defined by Equation (4):

(4) $$\chi^{\prime \prime }\left(\omega \right)\;=\;\chi (0)\;\cdot \;f(\omega /\omega_{p}\left(T)\right),$$

in which χ(0) is a constant and f(ω/ω_p_(*T*)), which describes the shape of the imaginary part of ε″ is a function of temperature *T*.

f(ω/ω_p_(*T*)) can be defined according to Equation (5):

(5) $$\omega_{p}\left(T\right)=A\;\exp(-w_{a}/kT),$$

in which $w_{a}$ represents the activation energy corresponding to dipole relaxation, A is a constant and *k* the Boltzmann constant (*k* = 1.38·10^−23^ J·K^−1^) [80].

A similar behavior of nanocomposites was reported in literature [117] and the relationship between the frequency and temperature at which the peak occurs was attributed to an Arrhenius-type behavior (Equation (5)). In the case of silica-filled LDPE nanocomposites (see herein above), the activation energy was determined using the Arrhenius-type equation (5) (Figure 23d–f).

The main relaxation in LDPE is α-relaxation attributed to the activation energy of dielectric relaxation (i.e., the orientation energy of the dipoles of the polymer chain and the segments of the polymer chain), which generally occurs at low frequencies. This activation energy is caused by dielectric relaxation and ranges between 1 and 1.5 eV [117]. The estimated activation energies of the silica-filled LDPE nanocomposites correlate well within this interval and increase with the nanoparticles content. If the nanoparticles concentration increases, more and more electrical dipoles will appear in the nanoparticles as well as in the polymer (main) chains and their lateral branches, resulting in more dipoles of the Si^δ+^→O^δ−^ and O^δ−^→H^δ+^ types within the three layers of the interface (Figure 20 and the model described in Section 5). These bonds are more flexible than the C^δ+^→O^δ−^ bonds and involve the movement of large fragments from the PE chain. If the nanoparticle concentration is doubled, the relative permittivity of the LDPE/nano-SiO_2_ nanocomposites should ‘mathematically’ as well increase by the factor of two. However, it has to be considered that the activation energy also increases with a rising concentration of nanoparticles (Figure 23d–f), thus the permittivity of LDPE/nano-SiO_2_ nanocomposites is lower. The variation of ε″ as a function of frequency and temperature in the case of nanocomposites of LDPE/SiO_2_ indicates the presence of an α-type relaxation process. This is confirmed by the shift of the peaks to higher frequencies with increasing filler concentrations and rising temperatures [108,111,118,119,120].

**Figure 24:**
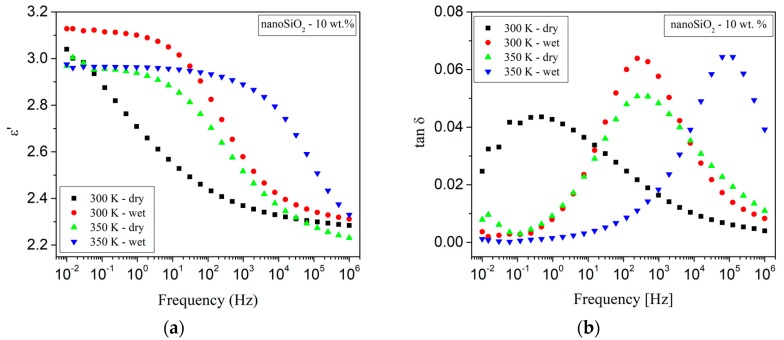
Frequency variation of (**a**) the real part of the complex relative permittivity and (**b**) the loss factor of LDPE/SiO_2_ nanocomposites with 10 wt % filler content at 300 and 350 K. Reprinted, with permission by the author, from reference [99].

Another study by Pleşa et al. on LDPE/10 wt % nano-SiO_2_ nanocomposites refers to the influence of moisture absorptionn and temperature treatment of the samples on ε′ and tanδ (Figure 24a,b) [99]. The results revealed that the drying step at elevated temperature has a crucial influence on the frequency corresponding to the tanδ peak. In particular at 300 K, the frequency corresponding to the tanδ peak is increased by four orders of magnitude (from approx. 0.2 Hz to 2 kHz) if the sample was kept in hot air before the dielectric measurements were carried out (Figure 24b). This distinctive increase can be explained by the high number of hydroxyl groups (O^δ−^→H^δ+^) on the surface of the nanoparticles (Figure 20 and the model described in Section 5). Hence, such types of nanoparticles are highly hydrophilic and contain adsorbed water molecules. These water molecules significantly influence the polarization phenomena even in very low concentrations [118]. Although LDPE is a hydrophobic material, the absorption of water molecules on the surface of SiO_2_ nanoparticles is thermodynamically inevitable [121]. By grating LDPE with maleic anhydride MA, hydrogen bridges are formed especially within the first layer of the interface area (Figure 20 and the model described in Section 5) between MA and the nanoparticles, as well as between the nanoparticle and the end groups of the polymer chains from the second layer of the interface. The energy of a hydrogen bond in a water molecule is about 21 KJ·mol^−1^; by storage of the nanocomposites samples at elevated temperatures before the measurements, the majority of adsorbed water molecules can be removed. Thus, the number of O^δ−^→H^δ+^ interactions is significantly decreased. Under these conditions, the energy consumed due the (re-)orientation of dipoles is lower, which corresponds to a decrease of the loss factor and a shift of dielectric relaxation to lower frequencies (Figure 24a,b).

Nanocomposites based on crosslinked polyethylene XLPE and silica nanoparticles are promising candidates for future power cables insulation due their improved dielectric properties [69,105,110,122,123]. The influence of moisture on the dielectric properties of these nanocomposites, however, has not yet been fully explored. Humidity is well known to be detrimental to dielectrics, reducing the breakdown strength and increasing the losses [25,26,27].

**Figure 25:**
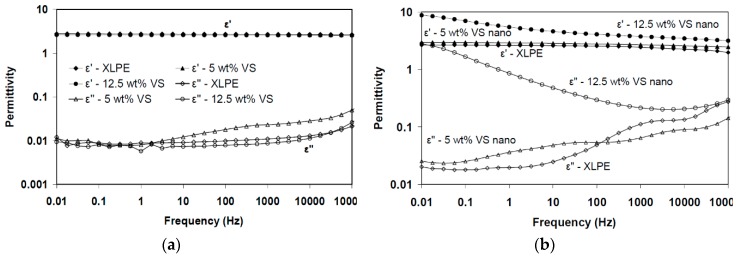
Correlation of permittivity and frequency for (**a**) dry and (**b**) wet samples of unfilled XLPE and nanocomposites filled with 5 and 12.5 wt % of vinyl silane-functionalized silica nanoparticles (storage of the ‘wet’ samples at 50 °C and 100% r.h.). Reprinted, with permission by IEEE, from reference [124].

Hui et al. [124,125] investigated the dielectric behavior of XLPE/silica nanocomposites in humid environments. Compared to the unfilled XLPE, nanocomposites containing silica particles were found to show increased moisture uptake due to filler inclusion. It was assumed that the dielectric behavior of wet XLPE/silica nanocomposites originated from the formation of water shells around the nanoparticles and the change of the inter-particle/cluster distances. The authors investigated unfilled XLPE as well as nanocomposites based on XLPE with unfunctionalized and vinyl silane-functionalized silica fillers (12 nm diameter) in concentrations of 5 and 12.5 wt %, respectively. A part of the samples was stored in humid environment for a month. Figure 25 a,b show the correlation of ε’ with the frequency for this set of samples. In the case of ‘dry’ samples, the permittivity in the low frequency region is very low, which originates from a lack of mobile charges (Figure 25a) [126,127]. For frequencies in the range from 10 to 100 kHz, an increase of the permittivity can be observed, which can be attributed to residual water [126,128,129]. In the case of wet samples (Figure 25b), loss peaks can be observed for nanocomposites with 5 wt % silica nanoparticles at frequencies in the range from 1 to 10^5^ Hz, and for unfilled XLPE in the range from 10^3^ to 10^5^ Hz, which can be attributed to water molecules present in the material. As silica nanoparticles have hydroxyl groups on their surfaces, they are likely sites to bind water; XLPE, on the other hand, is a non-polar material, in which the water molecules are more likely to be present in the amorphous regions, and the movement of dipoles can be inhibited by the structure of the polymer chains. Correspondingly, water present in XLPE triggers delayed dielectric relaxation. For the wet nanocomposites with 12.5 wt % of silica fillers, the significant increase of the real and imaginary permittivity at low frequencies might be explained by quasi-DC behavior [130] due to the ionic charge carriers [127].

When XLPE-based nanocomposites are exposed to humid environments, moisture is likely to dominate their dielectric behavior. Hui et al. observed that nanocomposites based on XLPE with 12.5 wt % silica absorb large amounts of water compared to unfilled XLPE and nanocomposites with 5 wt % of nanoparticles from the same humid environment [124,125]. This observation was assumed to originate from the percolation of water shells due to the higher concentration of nanoparticles and the correspondingly decreasing distance between them.

Numerous studies on the dielectric behavior of thermoplastic nanocomposites systems based on PE congeners like LDPE, HDPE and XLPE and different types (organic or inorganic) and concentrations of filler, usually between 0 and 10 wt %, were performed until present [14,131]. The most important question to be answered is whether or not the relative permittivity and loss tangent are reduced at the industrially relevant frequencies in nanocomposites, which could transform them into suitable candidates for power cables insulations. In literature, some reported data indicate the reduction of these parameters to certain extent, whilst other publications report the contrary [14]. These results can depend on many factors, such as how the particles were compatibilized or how the fillers were dispersed in the polymer, if the fillers agglomerated or not and so forth. Parameters such as humidity, temperature and so forth, have a significant effect as well.

## 7. Partial Discharges in Nanocomposites

### 7.1. Partial Discharges and Measurement Thereof

A partial discharge PD is a localized breakdown of a small portion of a solid or liquid electrical insulation between two conductors under high voltage stresses, which does not bridge the space between the conductors [132]. PDs occur inside cavities, cracks and/or gaseous inclusions inside solid insulations and at conductor-dielectric material interfaces in solid and liquid insulations. PDs with longer lengths deteriorate the insulation characteristics due to erosions of cavity walls by charge carriers, increases of the local temperature, radiation generated by atomic excitation and charge carrier recombination, intensification of the chemical degradation reactions, initiation of new chemical reactions and so forth. [133]. A general overview of erosion processes in thin polymer insulation under partial discharges is presented by Tanaka et al. [134].

The initiation and development of PDs depend on both, the shape and dimension of the cavities as well as the chemical nature and cavity gas pressure. On the other hand, electrical charges deposited by PDs on the cavity surfaces diffuse into the dielectrics (in the areas adjacent to the cavities) and form so-called space charge clouds, which dissipate slowly and change locally the distribution of the electric field.

**Figure 26:**
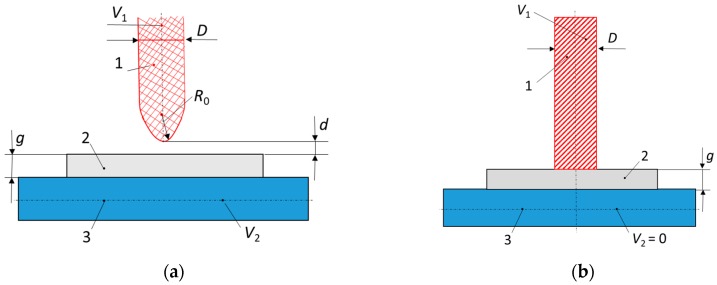
(**a**) Rod-plane electrode configuration for partial degradation tests: 1: Rod electrode; 2: Specimen; 3: Plane electrode; (**b**) Rod-plane electrodes system (similar to IEC Electrode) for surface erosion by PDs.

For the characterization of PDs, several variables are used, such as the PD inception voltage *U_i_* and the PD extinction voltage *U_e_*, the PD pulse repetition frequency *n* (average number of pulses in one second), the apparent charge *q* associated to the PD, as well as other derived quantities such as the apparent energy *W*, the average discharge power *P*, the average discharge current *I*, the quadratic rate *D* and so forth. [135,136]. For the study of the PD’s action on solid insulators, different types of set-ups are used, including comparably simple ones (Figure 26) that consist of a rod electrode from a tungsten wire with rounded tip, which is connected to the high-voltage terminal of an AC supply and a plane electrode of copper or steel that is connected to the ground, respectively to the supply terminal; the plain sample to be analysed is placed between the two electrodes (Figure 26a) [137,138,139].

Charge carriers erode the surface of the sample and the depth of the pits, the compounds formed on the eroded areas and the light emitted by the PD are parameters to quantify a sample’s resistance to PDs [139]. Experiments show that the PD initiation voltage decreases for larger cavities, while the maximum lengths, apparent energy and discharge power increase [140,141].

Considering the harmful effect of PDs, in the case of high and very high voltage cables insulation, restrictions are imposed on cavities dimensions and concentrations, such that their apparent charge does not exceed certain limits. For example, for a *U*_o_ = 110 kV voltage cable and a crosslinked polyethylene insulation, the apparent charge measured at a voltage of *U* = 1.5 *U*_o_ should not exceed 10 pC. On the other hand, PDs may occur at high voltage cable joints interfaces manufactured from two distinct insulating layers due to defects introduced during the technological process or during operation (i.e., metallic particles, fibers, cavities and layer depletions) [142].

In order to obtain insulation with the greatest possible resistance to the action of PDs, polymer nanocomposite materials have gained increased attraction. Nanofillers such as MgO, SiO_2_, Al_2_O_3_, rutile, layered silicate systems and so forth, increase the resistance against PDs due to multiple effects including nanoscale segmentation, permittivity difference, coupling agent and nanofiller pile-up [134]. The nanocomposites’ behavior to PDs is presented in numerous papers [134]. The majority of the studies focuses on the PD resistance of epoxy resins and polyimide nanocomposites. In the following section, some results are presented for the PD characteristics in PE-based nanocomposites used as power cables insulation.

### 7.2. Partial Discharges in Nanocomposites Based on Polyethylene (PE)

**Figure 27:**
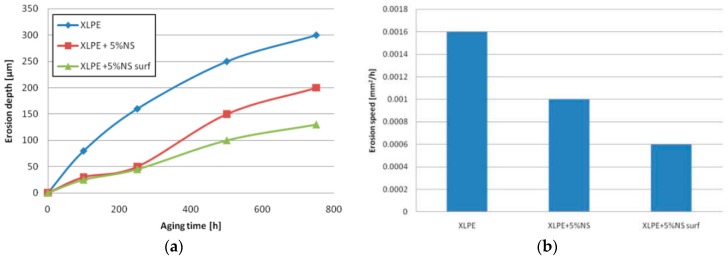
(**a**) Variation of the PD erosion pit depth with aging time and (**b**) average erosion speed values of unfilled XLPE, XLPE with 5 wt % of non-functionalized nano-SiO_2_ and XLPE with 5 wt % of surface-functionalized nano-SiO_2_. Reprinted, with permission by IEEE, from reference [105].

**Figure 28:**
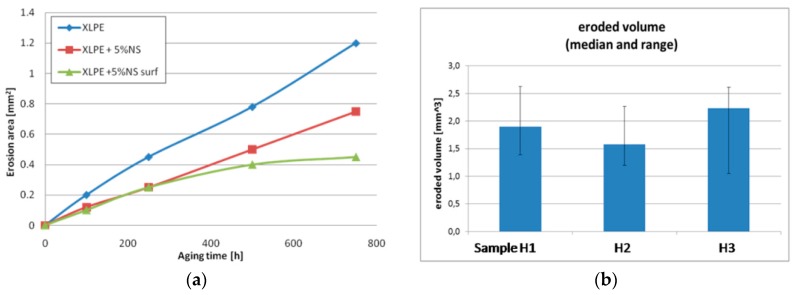
(**a**) Correlation of the cross-sectional area of pits with aging time for unfilled XLPE, XLPE with 5 wt % of non-functionalized nano-SiO_2_ and XLPE with 5 wt % of surface-functionalized nano-SiO_2_ [105]; (**b**) eroded volume by PDs produced with the IEC electrodes system type for unfilled XLPE (H1), XLPE with 5 wt % of non-functionalized nano-SiO_2_ (H2) and XLPE with 5 wt % of surface-functionalized nano-SiO_2_ (H3). Reprinted, with permission by IEEE, from reference [105].

The CIGRE Working Group D1.24 has conducted different tests in several laboratories from nine countries on the behavior of XLPE and its nanocomposite with fumed silica in an electric field [105]. The authors tested samples based on commercially available XLPE [105] in a set-up consisting of a rod-plane electrode and IEC electrodes systems (Figure 26). Three types of samples were tested: unfilled XLPE (XLPE–H1), nanocomposites filled with 5 wt % of unfunctionalized nano-SiO_2_ (XLPE + 5%NS–H2) and nanocomposites with 5 wt % of nano-SiO_2_ functionalized with a specific chemical coupling agent, selected to improve the dispersion in polyethylene (XLPE + 5%NS surf–H3). Correlations of the application time of voltage with the pit depth of the eroded area (at 10 kV_rms_, 250 Hz, 750 h) (Figure 27a), the average erosion speed (Figure 27b) and cross-sectional area of a formed pit (Figure 28a) were determined. It was found that samples with SiO_2_ fillers showed a higher resistance to discharges than those without fillers and that the highest resistance was found in composites with surface-treated fillers (Figure 27a,b and Figure 28a,b). Using the IEC electrodes system (Figure 26b), erosion in an area of 5 mm around the centre of the rod electrode was determined (for 50 h at a voltage of 10 kV_rms_ and 50 Hz) (Figure 28b). The results reveal that XLPE samples with untreated nanofillers show less erosion than the unfilled XLPE samples and that the erosion values for some of the XLPE samples filled with surface-functionalized nanofiller are higher (Figure 28b).

Using LDPE and fumed silica powder with a mean size of 7 nm, Aulia et al. studied the PD effect on these samples by positive and negative pulse counts using the CIGRE Method II (CM-II) electrode system [143]. The authors observed that the addition of nano-SiO_2_ in amounts of up to 4 wt % increased the number of PDs, while even larger concentrations of fillers in the range of 6 to 8 wt % significantly reduced the number of impulses. The same electrodes system (CIGRE Method II) was used by Sami et al. for the study of PD action on two types of PE-based nanocomposites, namely LDPE and HDPE (HDPE/SiO_2_ and LDPE/SiO_2_), with 0, 1, 2, 4 and 5 wt % of spherical SiO_2_ nanoparticles (15 nm in diameter with 99.9% purity and 14 nm in diameter with 99.9% purity) [144]. The authors measured the samples’ erosion depth and found that the values increased with an increased content of nanoparticles. It was considered that this behavior could be due to the defects introduced during the fabrication process of the samples [144]. Gao et al. showed that the use of montmorillonite MMT nanofillers (MMT = (Na,Ca)_0.3_(Al,Mg)_2_Si_4_O_10_(OH)_2_·n H_2_O) with high filling grades of SiO_2_ (more than 51 wt %) instead of spherical silica particles also increased the PD resistance of PE [145,146]. Both, the amplitude and the number of PDs were lower in PE/MMT samples than in unfilled PE (under the test condition applied) [145].

The influence of the MgO content on the PE resistance to PDs was analysed by Tanaka et al. [147]. The authors used the rod-to-plane electrode system (Figure 26) for flat samples of LDPE-based nanocomposites containing 0, 1, 5 and 10 wt % of spherical MgO particles with an average diameter of 50 nm. The results showed that the erosion depth of LDPE/MgO samples was significantly lower than that of unfilled LDPE samples (factors of up to 2.8). The authors explained the increase of the PD resistance of LDPE/MgO samples by the multi-core model, considering the fine segmentation of the polymer surface by nanofillers, the morphology formed around the nanofiller nuclei and the degree of bonding between the filler and the polymer [147]. The authors stated that the nanofillers were separated from the base matrix and piled on the surface.

The erosion induced by surface PD on samples of unfilled LDPE and LDPE filled with two types of inorganic nanofillers (quasi-spherical silica nanoparticles and synthetic layered MMT, each in 5 wt % content) was analyzed by Guastavino et al. [148]. The authors described that both types of nanocomposites exhibited longer lifetimes than unfilled LDPE under the same stress conditions.

Chen et al. [149] analyzed the effect of corona discharges on the performance of LDPE samples containing 0.5, 1, 3 and 5 wt % of nano-ZnO. The nanocomposites were exposed to the electric field for different time intervals. It was observed that a reduced content of ZnO nanoparticles lead to improved resistance to corona discharges. After 24 h of 10, 30 and 50 kV·mm^−1^ field application, LDPE/ZnO samples exhibited much lower values of accumulated space charge density than unfilled LDPE. The increase in aging time from 24 to 48 h reduced the volume resistivity and dielectric strength for all types of samples but these reductions were lower for LDPE/ZnO samples. In addition, the increase in the ZnO content caused an increase of the dielectric resistivity and breakdown strength of the samples, with a critical concentration of 5 wt %, at which these values decreased. The results suggested that the addition of nano-ZnO in low contents caused the occurrence of deep traps at the interfaces between nanoparticles and LDPE, in which space charges formed by discharges were accumulated. If the ZnO particle concentration amounted to 5 wt %, electrical resistivity and breakdown strength reached the maximum values after corona aging within the set of samples investigated. Zheng et al. [150] demonstrated that PDs degrade LDPE areas (in LDPE/ZnO nanocomposites) due to the combination of electrons associated with discharges and voids from the material, which generates UV irradiation.

A study of PD levels in HDPE was performed by Yamano and Okada [151] who added azobenzene derivatives such as azobenzene, *p*-nitro-azobenzene, *p*-amino-azobenzene and nitrobenzene-azo-resorcinol in amounts between 0.05 and 0.5 wt % to the polyolefin. The authors found that the level of PDs decreased by up to 20% compared to the samples without additives. It was concluded that this reduction relied on the prevention against secondary electron emission from the void wall due to the excitation of the azobenzene derivatives and prevention of electron detachment from the void wall due to the charge traps in the presence of the azobenzene derivatives [151].

The behavior of nanocomposites based on a blend of natural rubber (0–30 wt %) and LLDPE without or with 5 wt % of nanofillers of alumina trihydrate Al_2_O_3_·3 H_2_O was investigated using the CIGRE method II by Aulia et al. [152]. The results showed that the addition of natural rubber to LLDPE had positive effects on the PD resistance and that LLDPE blends with 20 wt % of natural rubber exhibited the highest PD resistance.

The studies presented in this section confirm that the use of polymer-based nanocomposites with organic or inorganic particles might lead to a reduction in PD degradation in power cable accessories and insulations, contributing to increased cables’ lifetimes. The increase in PD resistance of nanocomposites is due, among other things, to the following reasons:

1. Reduction of the polymer free space (preventing the erosion progress starting from PDs);
2. Segmentation of the polymer matrix (hindering the development of PDs);
3. Coupling agents that enhance the bonds between matrices and fillers (hindering the development of PDs);
4. Different values of the electrical permittivity of the matrix and the filler (decreasing the electric field local values and hindering the initiation and the development of PDs);
5. Nanofiller residues piled-up on the surfaces of specimens (hindering the development of PDs).

## 8. Space Charge in Nanocomposites

### 8.1. Space Charge Accumulation

Space charge is considered to be an excess of electric charge continuously distributed in a space region (volume or surface) and consists of electrons, holes and ions [153]. In terms of power cables, space charge is generally understood as a separation of free charge in the volume or interface of their insulation components due to: (*i*) carriers generated in the technological processes, (*ii*) space charge injection on the electrodes, (*iii*) the field-assisted thermal ionization of impurities from the insulation and (*iv*) the insulation degradation under the action of (electrical, thermal, mechanical, environmental etc.) stresses during operation [154]. In addition, space charge can accumulate in the case of the electric tree development [155,156] and/or in electrochemical approach (e.g., by water trees) [157,158,159,160,161]. With respect to DC power cable joints with multi-layered insulation, electric charges accumulate at the interfaces of the layers during operation due to the different values of the charge carriers’ relaxation time in the adjacent layers τ (Equation (6)):

(6) τ = ε/σ,

in which ε represents the permittivity and σ the electrical conductivity of the layer.

Space charge density is measured by different methods such as the piezoelectric-induced pressure wave propagation PIPWP method, the laser-induced pressure propagation LIPP method, the thermal step method TSM method, as well as the pulsed electro-acoustic PEA method [14,154,162,163]. The accumulation in time of the space charge contributes to the local intensification of the electric field, which accelerates the degradation process of the material [133,153]. Although the analysis of the global action of space charge on the insulation is difficult to achieve, it is still necessary to control and to reduce space charge accumulation in high-voltage DC systems [162].

### 8.2. Space Charge Reduction

Space charge accumulation in polymer cables insulation is closely related to the intensity of the electric field and the concentration of potential pits [111,164], the values of the free volume contained by them [165], the nature and states of the electrodes [154], the insulation dimensions [166] and the temperature gradient [167].

Fillers introduce additional trapping sites at particle interfaces and/or through morphological changes within the polymer matrix that serve to modify the original trap distribution [153]. As a result, the space charge accumulation in polymer nanocomposites changes compared to the unfilled polymers, depending on the nature of the polymer and the nanoparticles characteristics [168,169,170]. Thus, the volume density of the space charge has lower values [168] and its distribution changes in the case of nanocomposites compared to microcomposites [168,171,172].

Tanaka et al. demonstrated that the space charge inception threshold shifted to lower values for isotactic polypropylene i-PP-based as well as ethylene vinyl acetate EVA-based nanocomposites with nanofillers (from 12 to 4 kV·mm^−1^ for EVA and from 14 to 5 kV·mm^−1^ for PP) [111]. In addition, charges accumulated in the bulk at the field 60 kV·mm^−1^ tended to decrease with increasing addition of nanosized fillers from 2 to 6 wt % (for both, EVA and i-PP). Moreover, the charges dissipated more quickly in nanocomposites compared to microcomposites [153].

### 8.3. Space Charges in Nanocomposites Based on Polyethylene (PE)

**Figure 29:**
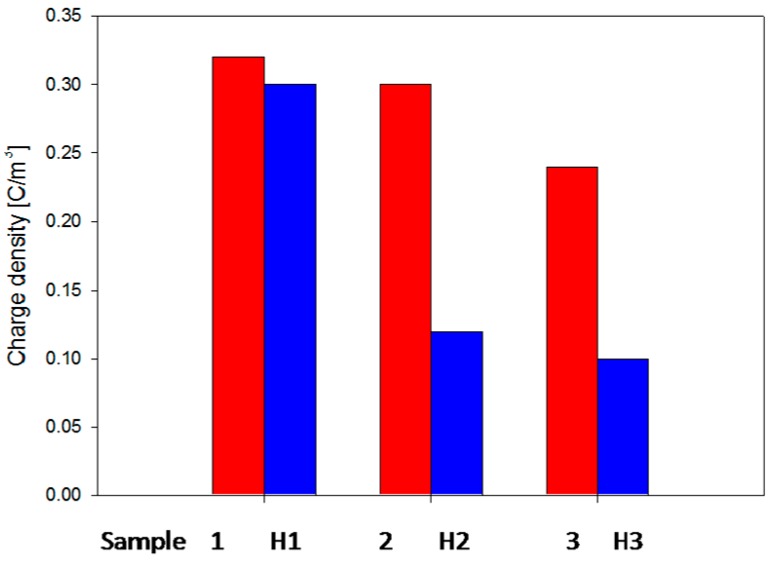
Space charge distribution at 20 kV·mm^−^^1^ in unfilled and filled XLPE prior to (red bars; samples 1, 2 and 3) and after treatment at 80 °C for 5 d (blue bars; samples H1, H2 and H3). Reprinted, with permission by IEEE, from reference [105].

The influence of fumed silica nanoparticles on the space charge accumulation in XLPE for power cable insulations was analyzed by the CIGRE Working Group D1.24 [105]. Three types of samples were used: unfilled XPLE (H1), XLPE with 5 wt % of unfunctionalized nano-SiO_2_ (H2) and XLPE with 5 wt % of nano-SiO_2_ surface-functionalized with a specific chemical coupling agent, selected to improve the dispersion in polyethylene (H3). The space charge was measured by the pulsed electro-acoustic PEA and the thermal step TS method. The results revealed that the lowest space charge amount occurred in composites with surface-treated nanofillers (Figure 29). The overall results showed that a heterocharge was generated in unfilled XLPE, which was associated with contaminant residues of the crosslinker and natural impurities [105]. Moreover, it was confirmed that nanofillers reduced this heterocharge as the nanoparticles were able to interact with the impurities. Concerning the charge injection, it was demonstrated that homocharges were injected more easily into filled than unfilled XLPE. It was also highlighted that charge packets appeared at very high electric field values [105] and that these charge packets were reduced by the addition of nanoparticles.

Lau et al. studied the influence of SiO_2_ nanoparticles, both untreated and treated with trimethoxy(propyl)silane, on the space charge formation in nanocomposites based on blends of LDPE (80 wt %) and HDPE (20 wt %) [153]. Space charge measurements were performed via the PEA method on samples subjected to electric fields of 25 and 40 kV·mm^−1^. The authors revealed that homocharges were separated near both electrodes and that their values increased with rising content of nanofillers. At 40 kV·mm^−1^, less charge accumulation was observed in samples containing surface-treated nano-SiO_2_ compared to composites filled with the same amount of untreated nano-SiO_2_ [153]. This behavior was explained by the accumulation of homocharges due to the generation of localized surface states upon the introduction of nanofillers. The influence of the electric field as well as the surface treatment of nano-SiO_2_ particles with diameters of 10 to 20 nm on the charge dynamics was studied by Wang et al. [173]. They used trimethoxy(propyl)silane-functionalized and untreated nano-SiO_2_ particles. The samples (blends of 80 wt % of LDPE and 20 wt % of XLPE; unfilled or filled with nano-SiO_2_) were subjected to DC high fields of 30, 50 and 70 kV·mm^−1^; the space charge was measured by the PEA method. The authors showed that a lower concentration of silica nanoparticles improved the dynamic space charge and that the composites with surface-treated particles had a better dispersion of the space charge. A reduction of the space charge was obtained in the presence of nanoparticles (especially homocharges) [173].

The influence of TiO_2_ on the accumulation of space charges in LDPE was analyzed in two studies [170,172]. Experiments were performed on LDPE samples with different contents of TiO_2_ of up to 6 wt %. It was demonstrated that: (*i*) for DC fields of 40 kV·mm^−1^, the hetero-polar space charge near electrodes was much lower in LDPE/TiO_2_ nanocomposites than in pure LDPE; (*ii*) the space charge inside the nanocomposites was more uniform in the nanocomposites than in pure LDPE (reducing local field strengths) and (*iii*) the space charge decreased after sample breakdown, which was faster in samples containing TiO_2_.

Xu et al. studied the influence of polyhedral oligomeric silsesquioxanes POSS nanofillers on the space charge accumulation in LDPE samples [174]. For tests, samples of pure LDPE and LDPE with 1 wt % of *iso*-octyl POSS (IOPOSS), octamethyl POSS (OmPOSS) and octa-*iso*-butyl POSS (OibPOSS) were prepared. In the absence of voltage, the homocharge density was higher in LDPE/OibPOSS and LDPE/OmPOSS samples (for voltages below 5 kV), while the charge injection in LDPE/IoPOSS under 10 kV was the lowest.

The effects of introducing nanosized BaSrTiO_3_ particles (contents of 2 and 10 wt %) into LDPE on the space charge accumulation in LDPE were studied by Fleming et al. [175,176]. By using 50 nm BaSrTiO_3_ nanoparticles, the authors prepared flat nanocomposite samples with heights of 150 to 200 μm with aluminium electrodes and subjected them to a DC voltage of 3.1 kV for 1 d at room temperature (r.t.). The results showed that the space charge density was approximately an order of magnitude smaller in the nanocomposite samples compared to unfilled LDPE.

The addition of ZnO nanoparticles in PE leads as well to changes in space charge values. The studies performed by Fleming et al. [95] demonstrated that the ZnO nanoparticles reduced the density of homocharge accumulation close to the electrodes. A study of the ZnO influence on the space charge in LDPE was performed by Tian et al. [177]. Using samples of LDPE with untreated ZnO (average size of 50 nm) at concentrations of 0.1, 0.5, 1, 3, 5, 7 and 10 wt %, the authors determined the space charge density for electric fields of 50 kV·mm^−1^. It was shown that the introduction of ZnO lead to space charge suppression in the nanocomposites due to a significant increase of the trap density.

Murata et al. studied the influence of MgO nanoparticles on the space charge accumulation in LDPE [178]. Flat samples with a thickness of 200 μm were prepared and subjected to DC fields of up to 125 kV·mm^−1^. The authors found that the amount of space charge was reduced in the nanocomposites. Furthermore, the ratio of stress enhancement was reduced by the addition of nanosized MgO-fillers in a certain range of concentrations: At the average stress of 125 kV·mm^−1^, the electric field enhancement ratio of LDPE was reduced by adding 1 wt % of nanosized MgO. However, if the MgO content exceeded 1 wt %, the values of the electric field enhancement ratio did no longer change. Similar findings were presented by Murakami et al. [98]. They observed that the increase in the amount of injected charge depended on the MgO content, which caused a reduction in space charge density values [98]. The space charge formation in LDPE/MgO nanocomposites under DC stress at high temperatures was studied by Taima et al. [179]. LDPE and LDPE/MgO nanocomposite films with a height of 100 μm and a MgO content of 0.2, 0.5, 1.0 and 2.0 wt % were prepared. In order to measure the space charge in DC fields of up to 200 kV·mm^−1^ and at elevated temperatures of up to 60 °C, an improved PEA system was used [180]. The study revealed that: (*i*) the introduction of MgO nanoparticles lead to space charge reduction, (*ii*) the space charge density increased with increasing field and temperature and (*iii*) the local enhancement of the electric field was reduced in LDPE/MgO nanocomposites, particularly at higher concentrations of MgO [180]. Peng et al. studied the space charge in films of 450 μm height based on LLDPE/MgO nanocomposites with a MgO content ranging from 0 to 5 wt % [19]. The samples were subjected to electric fields of 20 and 40 kV·mm^−1^ for 1 h at r.t. and the space charge was measured with a PEA system. The results revealed that, at low nanoparticle loading, the LDPE/MgO interfaces reduced the characteristic trap energy levels and bound charges injected from electrodes. Consequently, a reduction in space charge density in LDPE/MgO nanocomposites was achieved [19].

Research performed by Zheng et al. on LDPE/MgO nanocomposites showed that the space charge distribution improved and the decay rate of space charge increased with rising temperature [181]. The influence of the samples’ height and temperature gradient on the space charge accumulation in nanocomposites based on PE was analyzed by Chen et al. [167], Lv et al. [166] and Wu et al. [182]. Chen employed LDPE samples, while Lv was applying XLPE samples with 1 wt % of SiO_2_ fillers. In the case of unfilled XLPE samples, a correlation between the space charge distribution and the temperature gradient was obtained. For the XLPE/SiO_2_ nanocomposites, a correlation of the space charge density and the samples’ heights was observed: In the case of thin samples, heterocharges accumulated but their density decreased with the increase in samples’ heights. XLPE/SiO_2_ nanocomposites showed a more pronounced correlation of samples’ heights with the heterocharge density than unfilled XLPE under temperature gradients [166]. The influence of the nanoparticles’ size on space charge was studied by Wu et al. [183] on samples of LDPE/SiO_2_. The concentration of the SiO_2_ nanoparticles was kept constant at 2 wt %, while the particle sizes were varied among 7, 12 and 16 nm. The results revealed that the accumulation of space charge in LDPE could be suppressed by nano-SiO_2_ and that the nanoparticle content for which this effect was maximal depended on the size of the filler [183]. A similar study on the influence of SiO_2_ dimensions on space charge accumulation in LDPE was performed by Yin et al. [170]. The authors used SiO_2_ nanoparticles with dimensions of 7 and 16 nm and concentrations of 1, 3 and 5 wt %. It was found that the density of deep traps was significantly reduced after the introduction of nanofillers and that the density of deep traps is lower in nanocomposite filled with 7 nm nanofiller [170].

In the case of DC cables junctions with two distinct polymer layers (i.e., XLPE and EPDM), a layer of space charge is separated at their interfaces whose density depends on the conductivity and electric permittivity values of the polymer layers [133]. Replacing the EPDM layer with one containing SiC particles, the space charge accumulation on the interface gets reduced to significant extent, while the junction performance is improved [34].

## 9. Electrical Treeing in Nanocomposites

### 9.1. General Characteristics of Electrical Treeing

Electrical treeing is one of the main causes for failure and damage in electrical power cable insulations [184]. Electrical trees are networks of very thin channels (including gases at high pressures) initiated near insulation and/or electrodes defects, which develop in the insulation during the electrical equipment operation [133,185]. In order to study the electrical treeing and to define the resistance to trees, two methods based on the local electric field intensification are generally used, the needle-plane electrodes method and the electron beam irradiation method. The needle-plane electrodes method is employed particularly for studying the development of electric trees in the bulk of a sample, while the electron beam irradiation method is employed in particular for the study of surface trees [186].

**Figure 30:**
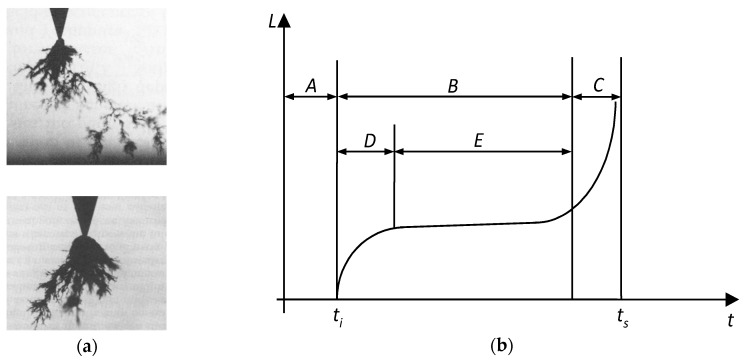
(**a**) Development of vented electric trees in LDPE. (**b**) Schematic representation of the electric tree development: A: inception phase; B: development stage; C: unstoppable growing (prior to breakdown); D: fast growing stage; E: slow propagation region; t_i_: initiation time (the time difference between the detection of electrical tree and the voltage application); t_s_: breakdown time (the time difference between the breakdown of the specimen and the voltage application). Reprinted, with permission by the author, from reference [133].

The electric tree channels (Figure 30a) are formed by the local destruction of the material under the synergetic action of the electric field, namely the PDs (from the cavities and channels), the heat and the mechanical stress produced by the electric field and the gases from the channels (yielded from material degradation). The electric trees initiated near the electrodes are called vented trees and they are developed from the electrode to the ’inside’ of the material (Figure 30a). In contrast, the trees generated inside the insulation (near cavities, metal inclusions, etc.) have a channels structure developed along the electric field lines on both sides of the defect (electric field concentrator) and are called bow tie trees. Electrical trees have been observed in all polymer classes used for medium- and high-voltage insulation: polyethylene [133], polypropylene [187], silicone rubber [188], ethylene propylene rubber [189], ethylene propylene diene rubber [190], polyimide [191], epoxy resin [192] and so forth. The electric trees obtained in PE with needle-plane electrodes in harmonic fields of 50 Hz are tree-shaped (or branched) for *E* < 5.4 GV·m^−1^, bush-shaped for *E* values ranging from 5.4 to 6 GV·m^−1^ and bush-tree-shaped for *E* > 6 GV·m^−1^ (these values decrease with increasing frequency) [42].

The development of electric trees is a discontinuous process, with sudden increases in trees’ dimensions (e.g., length *L*) followed by local breakdowns (Figure 30a,b). The involution period of the tree growth is considered as an inception (incubation) period for the occurrence of the ‘split’ within the initial branch (channel). During this period, PDs extend into the newly formed channels, contributing (by the energy transmitted to their walls) to the increase in diameters (and lengths of the last formed branches) [193]. This evolution is observed for both, tree- and bush-tree shaped congeners, up to the phase C, until the ’accelerated’ development phase starts (Figure 30b), in which *L* increases very fast until the thermal breakdown of the specimen has been reached.

Multiple studies of various types of PE for medium and high voltage cables insulations have highlighted the dependence of the electric tree rate development on various factors, such as intensity of the electric field [194], the electric field frequency [193,195,196], chemical characteristics [197] and physical structure of the polymer [198,199,200,201,202], temperature [194], impurities concentration [203], moisture content [204], mechanical stress [155,156,205,206,207], as well as electrodes characteristics, shapes and dimensions [208,209]. Several new PE compounds containing organic additives (such as polycyclic aromatic compounds or benzophenone derivatives) [203] were obtained, which can capture high-energy electrons and dissipate their energy [210] while forming aromatic anions [42]. Thus, depending on the properties and concentration of the additive, an increase in inception times and a reduction in the propagation rate of the electric trees in PE can be achieved [211]. In order to extend the lifetime of an insulation, composite materials with barriers and surrounding polymer matrix are commonly used in power engineering [212] and many research studies were performed on the development of nanocomposites for increasing electric breakdown resistance [14].

In recent years, a plethora of new polymeric materials has been developed, with improved properties compared to conventional polymers. These include copolymers and especially nanocomposites based on thermoplastic polymers and inorganic fillers [198,213,214]. These improvements depend on several factors, among which the properties of nanoparticles and nanoparticle-induced morphological changes at the nano- or micrometric scale are very important [198]. Based on the multi-core model (Figure 20) [100], an explanation of the increase in electrical treeing resistance of polymer nanocomposites was given [215]: It is considered that the treeing resistance of nanocomposites is due to the control of the acceleration of electron carriers by the diffusion layer and the multi-core layer. Namely, the electrical treeing resistance depends on the distance between the nanoparticles, the total area of the nanoparticle-polymer interface and, evidently, on the interface characteristics [215,216].

The electric tree growth in polymers can be slowed down by three methods: (*i*) changing the physical structure of the polymer [217], (*ii*) improving the manufacturing process by applying super-clean technology [218] and (*iii*) by adding voltage stabilizers [219]. In the presence of nanoparticles, the tree propagates through the polymer or the interfacial region and forms a zigzag pattern around the nanoparticles, thereby extending the pathway through the composite. Thus, the electrical tree needs more time to pave the way to the opposite electrode [185].

### 9.2. Electrical Treeing in Nanocomposites Based on Polyethylene (PE)

The increase of the electrical treeing resistance of PE by using organic additives was analyzed by Yamano and Iizuka [216]. The authors studied samples of LDPE, into which different aromatic additives such as naphthalene, anthracene, 9,10-dibromoanthracene, tetracene and pentacene were introduced. It was found that the inception voltage of electric trees *U_iet_* depended on the type of the compound added; anthracene was the most effective additive with respect to increasing *U_iet_* values.

Alapati et al. [198,220] studied the influence of alumina Al_2_O_3_ on the electrical treeing resistance, the inception voltage and the propagation rate of electric trees in unfilled LDPE as well as LDPE filled with 1, 3 and 5 wt % of nano-Al_2_O_3_. The authors observed that the introduction of Al_2_O_3_ nanoparticles into the polymer lead to an increase of the tree inception voltage with a filler content below 3 wt %. It was also described that the tree growth slowed down and changed in shape, namely from branch to bush, with rising filler loading. Both, the slow propagation of tree growth as well as the reduction in tree inception voltage at increased filler loadings, were attributed to the morphological changes of the nanocomposites. By scanning electron microscopy SEM, it was observed for higher filler loadings that the degree of crystallinity was reduced and the lamellae packing was increased. In addition, LDPE filled with 5 wt % of particles exhibited a high content of disordered spherulitic structures [198]. It should be noted that, for 5 wt % of Al_2_O_3_ content, the inception voltage of trees decreased. The authors explained this phenomenon by an increase in trap density resulting from the reduction of the critical field required for detrapping [220].

Yamano and Iizuka characterized the behavior of electrical treeing of nanocomposites based on LDPE and Al_2_O_3_ nanoparticles (0.5 to 9 wt %), phthalocyanine additives (0.5 to 8 wt %), as well as a combination of both fillers [216]. The authors showed that the exclusive addition of Al_2_O_3_ nanoparticles lead to insignificant increases of the inception voltage of the electric trees *U_iet_* only. They concluded that the increase of the *U_iet_* (in the presence of Al_2_O_3_ and phthalocyanine additives) was due to the effects of the π-electron resonance in phthalocyanine and the semi-conductive characteristics of phthalocyanine crystallites [215]. It was also shown that the time interval τ intervened between initiation of the tree in a sample and its breakdown was 10 times higher for the nanocomposites containing Al_2_O_3_ and phthalocyanine compared to unfilled PE. In samples containing only Al_2_O_3_ nanoparticles, τ was 3 times higher than in the unfilled polymer, while, in the case of LDPE samples containing phthalocyanines, τ was comparable to the unfilled polymer. The authors explained these results by a better dispersion of Al_2_O_3_ nanoparticles in the polymer matrix by the addition of phthalocyanines and the increase of the nanoparticle-polymer interface area. It is well-known that phthalocyanine is not soluble in LDPE and, thus, the additive forms small crystals with sizes in the range of 0.1 to several μm in the polymer matrix [221], which distorts the electric field. In order to avoid the effect of limitation in the inception voltage increase, Yamano prepared nanocomposites based on LDPE and Al_2_O_3_, in which phthalocyanine was replaced by an azobenzene derivative (having π-electron clouds with an absorption band of kinetic energy of the electrons from 2 to 5 eV) [151]. It was shown that for samples containing Al_2_O_3_ and 2 to 3 wt % of the azobenzene derivative, the initiation voltage of trees was 2.4 times higher compared to samples without additives. The duration of tree initiation increased in the case of samples with additives by approx. 100 times (for a voltage of 15 kV) for samples containing 3 wt % of Al_2_O_3_ and 2 wt % of the azobenzene derivative as additives. The use of Al_2_O_3_ also enabled to control the tree growth [221].

**Figure 31:**
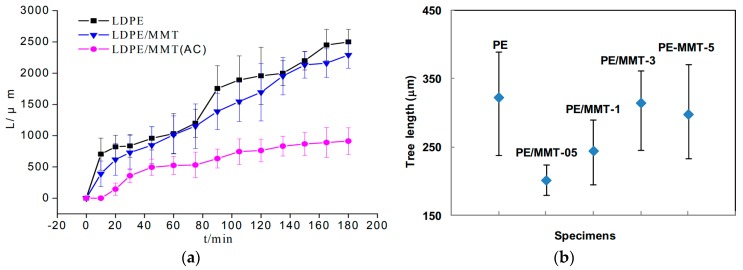
(**a**) Variation in time *t* of electric tree’s average lengths *L* developed in unfilled LDPE (LDPE), LDPE with 5 wt % of MMT (LDPE/MMT) and LDPE with 5 wt % of MMT treated in an alternating electric field (LDPE/MMT (AC)). Reprinted, with permission by IEEE, from reference [184]. (**b**) Length dispersion of electrical trees in PE and PE/MMT composites with varying content of MMT: 0 (PE), 0.5 (PE/MMT-0.5), 1 (PE/MMT-1), 3 (PE/MMT-3) and 5 wt % (PE/MMT-5). Reprinted, with permission by IEEE, from reference [223].

Addition of a certain content of montmorillonite MMT to PE improved the electrical treeing resistance of the material. This filler effect depended, among others, on the preparation approach and dispersion degree of MMT in the polymer (Figure 31a) [184]. It was also observed that the shapes of the trees were changed upon the addition of MMT. Tests were carried out on samples with dimensions of 10 × 8 × 3 mm using a needle-plane electrode system (tungsten-needle electrodes with a radius of 3 μm) at a voltage of 7 kV [222]. Branch-shaped trees with ‘non-conductive’ channels were obtained in unfilled LDPE, while bush-shaped trees with ‘conductive’ channels were observed in LDPE/MMT nanocomposites. In addition, the results revealed that the tree lengths were shorter by about 60% in LDPE/MMT nanocomposites compared to unfilled LDPE. The influence of the MMT dispersion morphology (isotropy in random arrangement or anisotropy in oriented arrangement) on the development of electric trees in LDPE was studied by Yang et al. [184]. The authors orientated the nanofillers in the matrix by applying an electric field that induced the field-controlled orientation, aggregation and arrangement of the nanoparticles. The sample morphology was influenced by the heating temperature, the type of electric field (AC or DC) and the intensity and application time of the field. It was observed that the applied electric field induced the orientation of the MMT particles parallel to the samples’ surfaces, hence achieving a uniform dispersion of MMT in LDPE. When the electric field was set for the tree initiation, the MMT particles were arranged perpendicular with respect to the direction of the electric field, acting as a barrier that inhibited electrical tree initiation (Figure 31a). The MMT barrier interrupted the growth and the original direction of tree growth [184].

In order to improve the electrical tree resistance of cable insulations, PE nanocomposites with nanoMMT layered fillers were prepared by Chi et al. [223]. The authors prepared plate samples with surface-modified MMT in concentrations ranging from 0.5 to 5 wt %. It was shown that the tree length *L_et_* in MMT/LDPE samples was reduced, particularly for lower filler concentrations. The lowest values of *L_et_* were obtained at a MMT concentration of. 0.5 wt % (Figure 31b) [223]. The development of electric trees in nanocomposites based on LDPE with 10 wt % of fibrous and laminar filosilicate nanofillers such as sepiolite (SEP10), montmorillonite (MMT10) and combined sepiolite and montmorillonite (MMTSEP10) was studied by Guastavino et al. [224,225]. It was found that the treeing inception voltage TIV amounted to 18.8 kV in unfilled LDPE samples. The values significantly decreased to 12.4 kV for SEP 10 and 15.8 kV for MMTSEP10, respectively, and remain practically unchanged at a value of 18.48 kV for MMT10 samples. It was also observed that the layered MMT platelets have the strongest effect on slowing down the propagation of electrical trees in LDPE nanocomposites [225].

Tiemblo et al. described the initiation and development of electric trees in LDPE with 5 wt % of spherical silica (SPH samples) and organically-modified fibrous phyllosilicate nanoparticles (FIB samples), as well as a modified laminar phyllosilicate montmorillonite (LAM) [226]. The modification of silica nanoparticles and the fibrous phyllosilicate was carried out with grafting strategies using methoxysilanes with *p*-toluenesulfonic acid as the catalyst. The time to inception TTI and the time to breakdown TBD of the samples were measured and the tree growth duration TGD was determined. The results showed that the tree shapes, TTI and TGD depended on the samples’ structures and the applied voltage. At voltages below 15 kV, the laminar silicate provided a barrier effect for the propagation of the electric trees as well as the initiation and propagation duration of the electric trees’ growth. This phenomenon was explained by the semicrystalline morphology of the nanocomposite upon the addition of LAM. Above 15 kV, however, this type of nanocomposite exhibited TTI values that were higher than those of unfilled LDPE. In agreement with literature data, this observation can be retraced to the interfaces of the large spherulites, which are populated with larger free-volume sites that promote the inception of electrical treeing [226]. The nanocomposites containing silica and the fibrous silicate did not show this barrier effect, as their geometry was either spherical (nano-SiO_2_) or fibrillar. Below 15 kV, the fibrous silicate did not influence the tree growth but caused an increase of their initiation time due to the particular semicrystalline morphology. With regard to nano-SiO_2_, a reduction in the inception of the tree duration and an increase in the tree propagation rate was observed [226].

The influence of MgO nanoparticles on the development of electric trees in LDPE samples was analyzed by Kurnianto et al. [227]. The authors used plate samples with filler concentrations of 0, 1, 5 and 10 wt %. The tree inception voltage TIV, breakdown voltage BDV, the tree development time *t_e_* and samples breakdown time *t_bd_* were measured. It was shown that the MgO nanoparticles acted as barriers for tree initiation and growth. The tree inception voltage increased with increasing filler content from 4.44 kV_rms_ for unfilled LDPE to 5.66 kV_rms_ for samples containing 10 wt % of MgO. Tree development times increased with increasing filler loading from 3.8 min for unfilled LDPE to 10.5 min for samples with 10 wt % of MgO [227]. These results were explained by the fact that MgO particles have a higher affinity for electrons than the polyolefin, which leads to easier electron trapping near the needle electrode. The initiation of some buds of a tree during tree development can be affected by trapping in localized states and local electric fields induced by nanofillers [228]. As a result, the formation of electron avalanches that initiate trees or generate new branches and develop new trees is hindered and the VIT increases. The propagation involves an electron collision with MgO particles, which prevents the development of an electron avalanche [227]. Thus, MgO particles slow down the initiation and the development of the electric trees.

Kawano et al. performed a similar study on the influence of MgO on the initiation and development of electric trees in nanocomposites [229]. The statistical analysis revealed that 63.2% of the respective tree inception voltages were 4.44, 4.60, 4.95 and 5.66 kV_rms_ for LDPE/MgO samples with 0, 1, 5 and 10 wt % of MgO content: The tree inception voltage increased with increasing nanofiller content [229]. Tanaka et al. tested LDPE/MgO samples with filler contents of 1, 2, 5 and 10 wt % at voltage frequencies of 60 and 600 Hz [230]. It was found that the inception voltage tended to decrease slightly if the filler content ranged from 0 to 2 wt % and increased slightly at higher particle concentrations from 2 to 10 wt %. Moreover, the tree length decreased with increasing filler content for less than 2 wt % of nanoparticles. At a filler concentration of 10 wt %, the tree growth is practically suppressed (Table 3) [230]. Using light emission to determine the inception voltage of trees in samples with relatively low contents of MgO nanoparticles, Tanaka et al. showed that the tree inception voltage was increased at a MgO concentration below 1 wt % (Table 4) [231]. It should be noted that the development of electric trees in polyethylene can also be achieved by electron beam irradiation without any applied field. A test performed by Xiao et al. on samples based on LPDE without and with nano-SiO_2_ showed that SiO_2_ nanoparticles reduced the tree lengths [232]. Also, the introduction of Buckminster fullerene (C_60_) or its derivatives such as [6,6]-phenyl-C61-butyric acid methyl ester (PCBM) lead to an increase of the inception field of electric trees by more than 25% [11].

## 10. Water Treeing in Nanocomposites

### 10.1. General Characteristics

**Figure 32:**
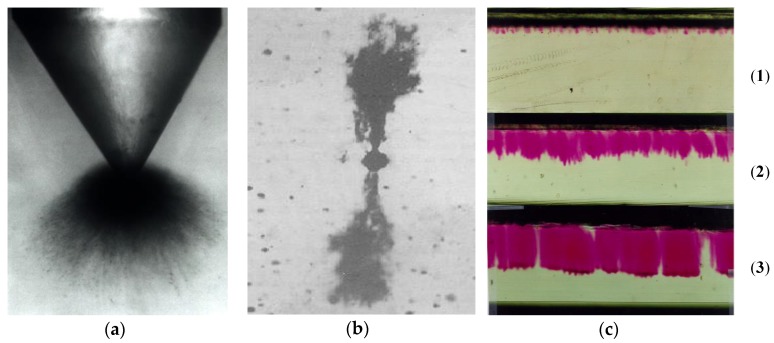
Water trees. (**a**) Vented tree developed at the tip of a needle electrode. (**b**) Bow-tie tree developed starting from an impurity. (**c**) Water trees developed in a laboratory from defects located at the surface of LDPE samples as a function of time t_1_ (1) < t_2_ (2) < t_3_ (3). Reprinted, with permission by the author, from reference [133].

Water trees are diffuse regions that appear inside insulations under the action of the electric field; they consist of water-filled microcavities. Water trees are formed along the electric field lines similar to electric trees. If the water trees start growing on the surface of electrodes, they are called vented trees. This is the case of trees growing in PE insulations of medium and high voltage electrical cables, where the growth starts from semiconductor layers in contact with needle electrodes (Figure 32a) and in plate samples with surface defects (Figure 32c) [133] and so forth. Bow-tie trees (Figure 32b) start growing at defects within the bulk material (impurities, cavities, etc.) and grow into the insulation. The typical characteristic of water tree branches is the formation of spherically shaped microcavities with a radius of 1 to 5 µm with a high density of 10^6^ to 10^9^ mm^−3^ [233]. Several parameters are used to characterize water treeing, namely the electric field and (inception) voltage, the length at a given time, the maximum length, the average growth rate, the area and the volume occupied by trees, the density of trees and so forth. [163] In water trees, Fe, Al, S, Na, K and C ions were detected in the bow tie trees and Cu, Si, S, K, Ca and Fe ions were detected in vented trees [133,234], as ions that drift with water molecules during the development of water trees and increasing content with the applied voltage [235].

Initiation and water tree growth occur particularly in the lower density regions of insulations (amorphous regions) [236] and are influenced by several factors such as the intensity and frequency of the electric field [42,133], the characteristics and concentration of the salts in the liquid that is in contact with the insulation [237], the concentration of additives [238], the temperature [239,240], the degradation degree of the material [239,241], the quality of the electrode surfaces and semiconductor layers and so forth. [242].

Notingher et al. showed that a critical field strength exists for each material [243]; above that threshold, water trees are formed. Various types of mechanisms have been proposed for the initiation and growth of water trees. Overviews were given by Nunes and Shaw [244], Shaw and Shaw [245], Steennis and Kreuger [236], Dissado and Fothergill [42], Ross [242], Crine [239], Notingher [133] and so forth. It can be concluded that two types of mechanisms seem to play a decisive role in water trees growth: (*i*) mechanisms based on the electrochemical degradation of the insulation and (*ii*) mechanisms based on the mechanical action of the electric forces on the material [246]. A detailed analysis of these mechanisms can be found in references [42] and [133].

Water trees in insulations lead to significant deterioration of the electrical properties and to local increase of the electric field [247]. A reduction of resistivity was observed in various studies by Bahder et al. [248], Tabata et al. [249], Tanaka et al. [250], Steennis et al. [236], Wojtas [251] and so forth. As the water concentration increases in the “tree regions”, the permittivity increases correspondingly compared to the material without water trees [252]. The measurements performed by Stucki on samples of PE insulation from cables containing water trees revealed a relative permittivity between 2.7 and 3.6 [253]. Also, dielectric losses increase in insulations deteriorated with water trees. This phenomenon has been studied on samples manufactured in laboratory scale [254] and on insulations of cables in operation [255].

**Figure 33:**
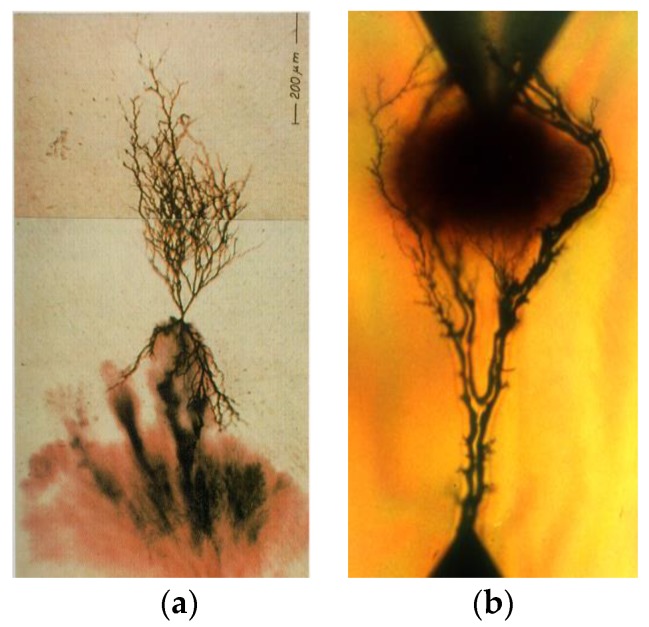
Electric trees developed in the presence of water trees. (**a**) The electric tree starts growing from the water tree. (**b**) The electric tree circumvents the water tree. Reprinted, with permission by the author, from reference [133].

Water tree development reduces the insulation breakdown strength (Figure 33a). If electrical trees are initiated in regions with defects such as cavities, impurities and so forth, these trees will develop around the region of the water trees until the insulation breaks down (Figure 33b). In order to reduce the number and growth rate of water trees, retardants such as organometallic compounds, additives with hydrophilic groups and so forth, are added to polymers, providing, for example, tree-retardant XLPE (TRXLPE) [256,257,258]. Over the last years, insulations based on various PE-based nanocomposites were manufactured. For the experimental work, two types of models are mainly used, (*i*) the uniform field models [246,259] and (*ii*) divergent field models [246,260,261]. The electrolyte solutions are commonly prepared with NaCl but also other salts such as AgNO_3_, CuSO_4_, Fe_2_O_3_ and so forth, are used; their concentrations being in the range of 0.01 to 10 M [246].

### 10.2. Water Treeing in Nanocomposites Based on Polyethylene (PE)

**Figure 34:**
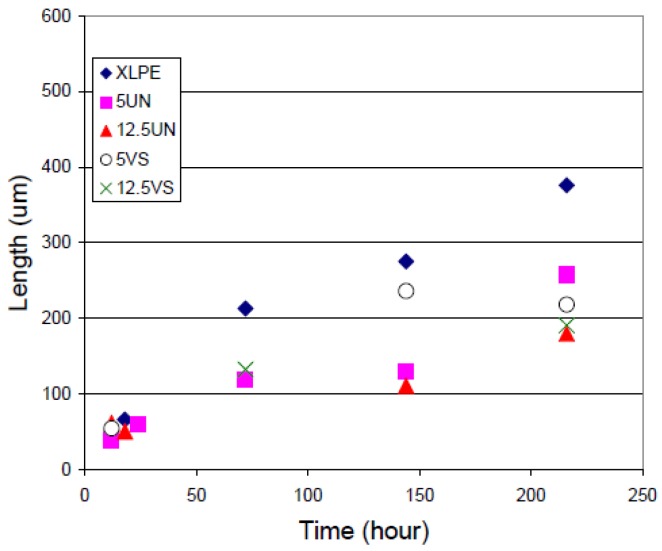
Variation on time of the water tree lengths in unfilled XLPE (XLPE) and composites with untreated nano-SiO_2_ fillers (5UN and 12.5UN) or congeners treated with vinyl silanes (5VS and 12.5VS). Reprinted, with permission by IEEE, from reference [260].

Series of research studies were performed on samples based on LDPE and XLPE with different types of inorganic nanofillers [240,260,262,263,264,265]. Huang et al. studied the effect of nano-SiO_2_ particles on the water trees growth in LDPE [262]. LDPE samples without filler (LDPE) were compared with nanocomposites containing 2 wt % of untreated Aerosil 200 nano-SiO_2_ (LDPE 200) and with samples containing the same amount of surface-modified nano-SiO_2_: For surface modifications, either octyl-trimethoxysilane (LDPE-O) or dimethyldichlorosilane (LDPE-D) were used. For the production of water trees, the authors used plate samples the sandblast surfaces of which were in contact with the electrolyte (1.0 M NaCl solution). A voltage of 5 kV with a frequency of 50 Hz was applied for 45 d and the tree lengths *L_wt_* were measured as 827 μm for LDPE, 601 μm for LDPE-D, 573 μm for LDPE-O and 531 μm for LDPE 200. This study revealed that nano-SiO_2_ could suppress the water tree growth in PE. In addition, it was shown that hydrophilic SiO_2_ comprised a higher efficacy in reducing the rate of water trees growth compared to hydrophobic SiO_2_, which can be explained by the alternation of the LDPE morphological structure due to the presence of nano-SiO_2_ and by the generation of small spherulites, in which water trees developed faster [266]. Huang et al. [263] conducted a similar study using samples based on LLDPE without fillers and with nano-SiO_2_ in 2.5 wt % quantity, the surfaces of which were either untreated (LLDPE-U) or treated with octyl-trimethoxysilane (LLDPE-OCT). After 45 d of sample aging, water trees developed in each sample. It was observed, however, that the water tree growth had lower values in the samples with fillers than in unfilled LLDPE. The authors attributed this behavior to the increase of the number of small spherulites upon the introduction of nanoparticles and the hydrophilic character of the untreated particles. Stress tests with high frequency voltages of 1 kHz using a water needle-plane geometry were performed by Hui et al. on XLPE samples [260]. The authors used plate samples based on XLPE without filler (XLPE) and with nano-SiO_2_ fillers in 5 or 12.5 wt % quantity. Two types of nanocomposites were prepared: either with untreated nano-SiO_2_ fillers (5UN and 12.5UN) or with vinyl silane-treated nano-SiO_2_ fillers (5VS and 12.5VS). Using a NaCl solution with a concentration of 0.5 M, it was confirmed that the morphology of water trees changed by the addition of SiO_2_ nanoparticles. In neat XLPE samples, trees grew preferentially from the water needle electrode towards the ground plane, while, in nanocomposites, the water trees had a wider fan-shaped morphology [260]. Furthermore, the nanocomposite with functionalized particles showed a decrease of water tree growth compared to the pristine XLPE (Figure 34). With the increase in the nanoparticle content, water tree development was further restricted. These observations were attributed to the interfacial regions introduced by nanoparticles. In the case of functionalized nanoparticles, more robust interfacial region was argued to be obtained, which further enhanced the resistance to the development of water trees [260].

The influence of MMT on the development of water trees in XLPE was studied by Li et al. [264]. The authors carried out experiments on plain XLPE samples with a height of 20 mm and nanocomposites with MMT in quantities of 1, 2 and 3 wt %. It was observed that the length of water trees decreased in the nanocomposite samples. This decrease was more pronounced in samples with higher filler contents and ranged from 259 μm in XLPE samples to 107 μm in XLPE/MMT samples with 3 wt % of MMT. This observation was explained by the MMT barrier effect and by the structure of the layered-silicate: The layered structure of MMT was argued to change the electric field distribution and to reduce the pitch of the needle, which slowed down the development of the water trees. The ionic bonds between (defects in) PE and the layered silicate molecules were held responsible for decreasing the relaxation of polymer segments, which reduced the dispersion of the water molecules and slowed down (or even prevented) the formation and growth of water trees [264].

The effect of MgO nanofillers on the water tree retardancy in LDPE and XLPE was characterized by Nagao et al. [240]. The authors used plate samples without and with MgO nanofillers. Water trees were produced in a uniform field, in 1 M of aqueous NaCl solution, an AC voltage of 5 kV_rms_ with a frequency of 400 Hz and temperatures of 313 and 333 K for 46 to 192 h. It was found that the development of water trees in nanocomposites was slowed down and that this effect increased with increasing nano-MgO content. At MgO contents of less than 2 wt %, the density and the length of the trees were lower in MgO/XLPE compared to MgO/LDPE samples.

It can be concluded that the introduction of inorganic nanoparticles into PE results in a slow-down of the water treeing phenomena. However, there are still aspects unclear regarding the explanation of retardant effect in PE, as well as the effect of nanoparticles in other polymers used as power cable insulation (EPR, silicone rubber, etc.).

## 11. Electrical Breakdown of Nanocomposites

### 11.1. General Aspects of the Breakdown in Solid Dielectrics

The breakdown of a dielectric occurs at a high concentration of charge carriers such as free electrons in the conduction band and/or holes in the valence band, particularly in the specimen transition in a conductive state [133]. Macroscopically, the breakdown involves a sudden increase in the conduction current passing through an insulator. The minimum value of the electric field strength, at which the breakdown of the dielectric occurs, is called breakdown strength *E_bd_* and the minimum voltage applied to the electrodes is called breakdown voltage *U_bd_*. The values of dielectric breakdown depend on many factors such as chemical nature of the polymer and its physical structure, the specimen dimensions, the temperature, the frequency, the duration of the applied electric filed, humidity, type and form of electrode and so forth. Depending on the physical processes that cause the breakdown of a solid insulating material, breakdown mechanisms are classified as thermal, electronic, electromechanical, free-volume, by PDs and by treeing (electrical and electrochemical).

Generally, the breakdown strength of the polymer depends on its morphology. In intraspherulitic regions, *E_bd_* is higher than in interspherulitic regions and a change in the disorder within the spherulites and/or the interspherulitic region can affect the voltage endurance and, of course, the breakdown strength [13]. Since the nominal voltages of the power cables are increasing (exceeding 500 kV), polymer materials with a higher breakdown strength are required for insulation. The incorporation of inorganic nanofillers into insulation can increase the *E_bd_* of the materials, depending on the filler concentration, their shape, size and surface modifications with different agents, material homogeneity, electrical properties of the fillers, sample preparation routes and so forth [267,268,269].

### 11.2. Electrical Breakdown in Nanocomposites Based on Polyethylene (PE)

The performance of PE/SiO_2_ nanocomposites in high electric fields is presented in numerous papers. A rather detailed experimental study was carried out by CIGRE WG D1.24 [105]. The authors used plate samples of pure XLPE and XLPE with 5 wt % of fumed nano-SiO_2_ fillers, either unfunctionalized or surface-functionalized by a specific chemical coupling agent [105]. Some of the samples were heated at 60 °C in vacuum for 8 d. Three types of cells were used to perform the tests, namely with spherical and cylindrical electrode systems for AC breakdown tests and with needle to plane electrode system for impulse voltage tests. It was observed that (*i*) the addition of nanofillers enhanced the AC breakdown strength, (*ii*) the functionalized nanofillers seemed to cause only a slight increase of *E_bd_*, (*iii*) heat treatment seemed to increase the *E_bd_* values, and (*iv*) correspondingly, the highest values of *E_bd_* were obtained in samples with surface-functionalized nanofillers after heat treatment.

**Figure 35:**
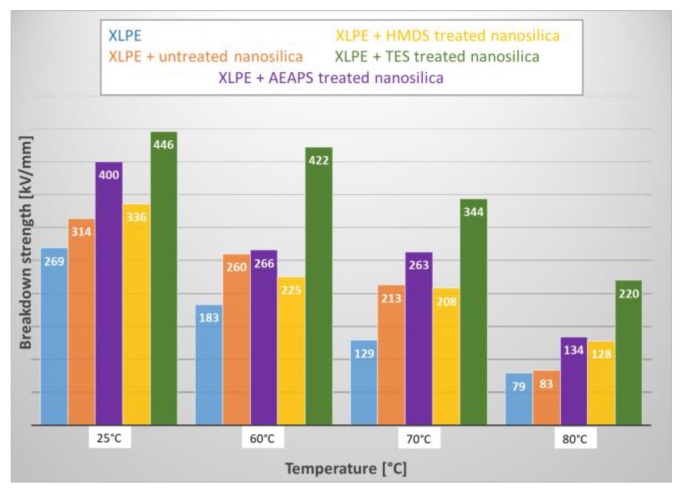
Breakdown strength for different XLPE nanocomposites (KV·mm^−^^1^). Redrawn and adapted from reference [13].

A comparative experimental study on the influence of silica micro- and nanoparticles on breakdown strength and voltage endurance of PE was performed by Roy et al. on plate samples of XLPE and SiO_2_/XLPE [13,122,270] (Figure 35). As filler, 5 wt % of nano-SiO_2_ were used, either untreated or surface-modified with triethoxyvinylsilane (TES), *N*-(2-aminoethyl) 3-aminopropyl-trimethoxysilane (AEAPS) or hexamethyldisilazane (HMDS). It was shown that (*i*) there was a significant increase in breakdown strength for nanofilled composites compared to unfilled XLPE, (*ii*) the samples with modified nano-SiO_2_ had a more significant increase in *E_bd_* compared to the untreated ones, (*iii*) the TES nanocomposite samples that were stored at 80 °C exhibited the highest breakdown strength; (*iv*) the breakdown strength had lower values in micro- than in nanocomposites. A similar finding was also reported by Reading and Vaughan [271]. In order to explain the increase of DC breakdown strength and voltage endurance of the nanocomposites compared to the unfilled and microfilled composites, the authors stated that the large number of small particles acted as scattering centres and that the nanoparticles altered the crystalline morphology of XLPE (providing another scattering mechanism). It was suggested that the nanoparticles within the crystalline phase could disturb the continuity of the path provided to the charge carriers, which increased the breakdown strength values. Furthermore, the smaller values of *E_bd_* in microcomposites (compared to nanocomposites) were argued to be due to the higher number of defects in microcomposites [272]. In order to explain the existence of higher *E_bd_* values in nanocomposites due to surface functionalization of nanoparticles, Roy argued the emergence of ‘quasi-conductive’ layers [273] in nanocomposites with untreated particles that locally reduce the electric field and do not appear in composites with surface-treated nanoparticles. In addition, polar surface treatments (by, for example, AEAPS and HMDS) have very deep trap sites, which reduce the mobility of charge carriers and increase the breakdown strength [106]. This explanation also takes into account the increase of the interfacial area and the reduction of the free volume in nanocomposites [122,270]. In the case of some polymers, the change in free volume due to the introduction of nanofillers is relatively low and does not excessively affect the breakdown strength [274].

**Figure 36:**
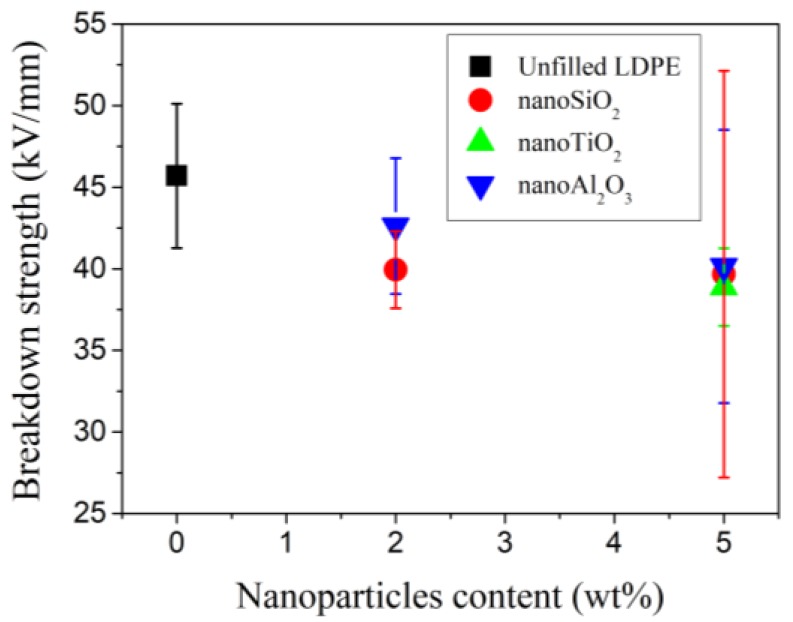
Values of breakdown strength of nanocomposites based on LDPE. Reprinted, with permission by the author, from reference [99].

Pleşa studied the influence types and contents of inorganic nanoparticles on the performance of plane-shaped LDPE-based nanocomposites in high fields [99]. As inorganic fillers, SiO_2_, Al_2_O_3_ and TiO_2_ were used (Figure 36; Table 5). The highest breakdown strength was obtained with LDPE/SiO_2_ samples that showed comparable breakdown strength of approx. 40 kV·mm^−1^, which, however, were lower than those of unfilled LDPE (~ 46 kV·mm^−1^). In general, the decrease of the breakdown strength in LDPE nanocomposites with inorganic nanoparticles is quite low, which could be correlated with the change in the degree of crystallinity in the nanostructured materials.

Lau et al. studied the influence of nano-SiO_2_ with particle sizes of 10 to 20 nm on the breakdown strength of blends containing 80 wt % LDPE and 20 wt % HDPE [153,275]. Tests on the AC and DC breakdown behavior of PE blends upon the addition of different contents of nanofillers (2, 5 and 10 wt %) and surface modifications (untreated particles and particles surface-treated with trimethoxy(propyl)silane were performed [275]. The addition of 2 and 10 wt % of untreated nano-SiO_2_ reduced the DC *E_bd_* from 480 (unfilled polymer) to 278 and to 307 kV·mm^−1^, respectively. In composites with surface-treated nano-SiO_2_, the DC breakdown strength was higher than that of composites with untreated nanoparticles. For samples with 2, 5 and 10 wt % of treated nano-SiO_2_, the *E_bd_* values were 58, 55 and 21 kV·mm^−1^ higher than those with untreated nano-SiO_2_. In all cases, *E_bd_* of the filled PE was lower than that of the unfilled analogue [275]. The authors explained the reduction of *E_bd_* by the accumulation of space, the increase in charge mobility and nanoparticle agglomeration effects [153]. Tests performed in AC showed a slight reduction of *E_bd_* values in samples with a filler content of 5 wt % of untreated nano-SiO_2_ and a reduction of over 30 kV·mm^−1^ for those containing 10 wt % of filler compared to the unfilled samples [275]. In the case of samples containing surface-treated nano-SiO_2_, the AC *E_bd_* values were higher than those of non-filled samples, particularly if the nano-SiO_2_ content amounted to 10 wt %. In those samples, the sizes of agglomerates were much smaller, which could be the reason for the improved AC breakdown strength [275].

**Figure 37:**
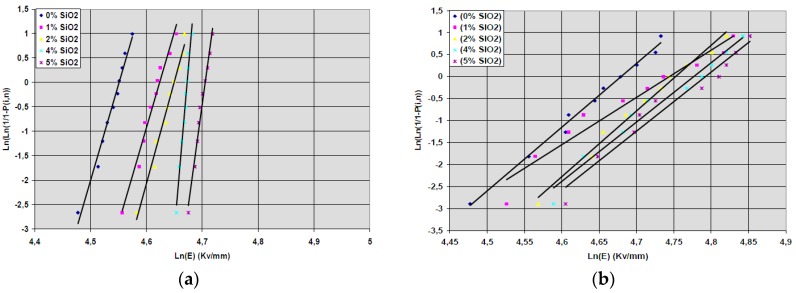
Weibull plots of breakdown strength of neat and nanocomposites with varying content of SiO_2_ nanoparticles based on (**a**) LDPE and (**b**) HDPE. Reprinted, with permission by IEEE, from reference [144].

The influence of the matrix structure and the nanofiller content on the breakdown strength of nanocomposites was described by Sami et al. [144]. For the tests, the authors used plate samples with a height of 250 μm based on nanocomposites of the composition HDPE/SiO_2_ and LDPE/SiO_2_ with 0, 1, 2, 4 and 5 wt % of spherical nanoparticles. The cumulative probability of the electrical failure *P* was analysed using the two-parameter Weibull function (Figure 37) [276]. *E_bd_* increased with increasing nanofiller content, both for LDPE as well as for HDPE nanocomposites. The highest *E_bd_* values were obtained for HDPE/SiO_2_ with 4 to 5 wt % of nanoparticles [144]. The dielectric strength growth was measured in AC on LDPE/SiO_2_ and XLPE/SiO_2_ nanocomposites in comparison with the unfilled PE and was reported in additional publications [277,278].

The influence of humidity on the breakdown strength of nanocomposites was studied by Hui et al. on XLPE nanocomposites with unfunctionalized (UN) and vinylsilane-functionalized (VS) SiO_2_ fillers [123] at loadings of 5 and 12.5 wt % [125]. Multi-recess samples were prepared and exposed to a humid environment of 100% r.h. and 75% r.h. at 50 °C for 14 d. The nanocomposites had a much higher moisture uptake compared to the crosslinked PE resin [125]. The presence of nanoparticles caused an increase of the breakdown strength; under humidity and heat, the *E_bd_* values were reduced for all types of samples. In some tests of the nanocomposites, lower *E_bd_* values were obtained than in unfilled PE [153,173]. This may be due to the space charge accumulation inside the samples and the local enhancement of the electric field [153].

The change of breakdown strength in samples based on LDPE filled with MMT and MA was presented by Gao et al. [145]. It was shown that the introduction of MMT increased the breakdown strength 1.35 to 1.70 times. The authors explained the increase of *E_bd_* of the nanocomposite by the increase of polarity (MA) and the decrease of the free volume (MMT). Both processes intensify the electron scattering and decrease the initiation probability of a breakdown channel. Using nanocomposites based on LDPE and the MMT cloisite 15A in 5 wt % quantity, Guastavino et al. noted an increase of the breakdown strength as well [279]. Green et al. investigated samples of a blend of a high density linear polyethylene HDLPE (10 wt %), a branched low-density polyethylene LDBPE (90 wt %) and a PE/MMT masterbatch MB, which contained 40 wt % of MMT functionalized with dimethyldi(hydrogenated tallow) quaternary amine [280]. The introduction of masterbatch MB led to an increase in dielectric strength from 143 ± 9 MV·m^−1^ (in the absence of MB) to 171 ± 3 MV·m^−1^ (in the case of 20 parts MB) [280]. A very important role in manipulating the breakdown strength is the way how samples were prepared. If the dispersion of MMT nanoparticles into the polymer matrix was inhomogeneous, the values of the breakdown strength were considerably reduced compared to samples with very well dispersed nanocomposites [281,282]. Shah et al. studied the influence of the MMT content on the breakdown strength of nanocomposites based on HDPE [283]. The authors prepared samples of HDPE with MMT modified by hexadecyl trimethyl ammonium bromide, untreated or treated with 3-aminopropyl triethoxy silane and titanium acetylacetonate in quantities of up to 10 wt %. The results revealed that the breakdown strength of the nanocomposites increased with increasing clay content of up to 5 wt %. If the concentration exceeded 5 wt %, the breakdown strength slightly decreased. The *E_bd_* values were also influenced by the type of nanoparticles and amounted to 30 kV·mm^−1^ for unfilled HDPE, 48 kV·mm^−1^ for HDPE with unmodified MMT and 54 kV·mm^−1^ for organoclay nanocomposites [283]. These changes were explained by the fact that exfoliated and intercalated clay platelets altered the electric field repartition and increased the path length for the breakdown.

The use of POSS as filler in LDPE yields composites with breakdown strength different from that of the LDPE matrix. Guo et al. demonstrated that the breakdown strength decreased if octamethyl POSS was applied in 5 wt % quantity, whilst the use of *iso*-octyl POSS in the same amount lead to a decrease of the breakdown strength [284]. The introduction of 0.3 wt % of functionalized graphene within LDPE improved the *E_bd_*, while the addition of graphene oxide did not provide any significant change at a content of up to 0.3 wt %.

In a series of papers, the influence of MgO nanofillers on the breakdown strength of PE is detailed. Reddy et al. [285,286] studied the DC breakdown strength of LDPE nanocomposites with small amounts of MgO nanoparticles. Maximum thermal voltage MTV calculations, also known as thermal breakdown voltage, were carried out. The results showed that the values of the MTV increased with nanofiller contents of up to about 5 wt %, for which *E_bd_* exhibited a maximum and decreased afterwards. Similar results have been obtained by Tanaka et al. [230], Masuda et al. [97], Murakami et al. [98] and Murata et al. [178]. Peng et al. used plate samples based on LLDPE and MgO nanoparticles surface-modified with (3-aminopropyl) triethoxysilane in concentrations of 0.1, 1, 2 and 5 wt % [19]. The results revealed that, at low nanoparticle loading, the LLDPE/MgO interface produced a large number of shallow traps, which increased the LDPE/MgO interface traps in the nanocomposites. Thus, the number and energy levels of free charges was reduced, enhancing the nanocomposites’ breakdown strength. The introduction of 1 wt % of MgO nanoparticles into LLDPE increased the breakdown strength from 346.8 to 380.2 kV·mm^−1^ [19].

The introduction of TiO_2_ into PE also increases the breakdown strength. This effect was highlighted by Ma et al. [287] who conducted a study on LDPE-based nanocomposites. The presence of water on the nanoparticle surfaces decreased the *E_bd_*: Samples prepared with dried nanoparticles exhibited 50% higher values than those filled with as-received TiO_2_ nanoparticles. Samples filled with surface-functionalized TiO_2_ showed an approx. 40% increase of *E_bd_* [287]. The surface modification was carried out with *N*-(2-aminoethyl) 3-aminopropyl-trimethoxysilane (AEAPS) as a coupling agent.

An experimental study by Tian et al. on plate samples of LDPE filled with untreated ZnO nanoparticles with a mean diameter of 50 nm showed that the breakdown strength of the nanocomposites with a content of less than 1 wt % was higher than those of unfilled PE [177]. If the content was higher than 1 wt %, the *E_bd_* values were lower than in unfilled LDPE [177].

From the studies described above, it can be concluded that nanoparticles can influence the breakdown strength of nanocomposites by different mechanisms such as (*i*) serving as heterogeneous nucleation agents (that accelerate the formation of crystalline areas and prohibit the formation of large spherulites), (*ii*) becoming scattering sources for electrons, (*iii*) decreasing the mobility of electrons by forming a large amounts of charge traps, (*iv*) hindering the polymer from the erosion of partial discharge, (*v*) inducing large void defects as well as distorting the local electric field (due of the large permittivity of nanoparticles), (*vi*) forming conductivity pathways and (*vii*) increasing the tunnelling current between nanoparticles.

As was mentioned above, the introduction of inorganic nanofillers into PE leads to the improvement of its electrical properties. On the other hand, some nanoparticles such as SiO_2_, ZnO and MgO cause an increase in the aging resistance (respectively, an increase in lifetime) of the nanocomposites compared to unfilled polyethylene [288,289].

## 12. Conclusions and Outlook

Due to the internationally increasing demands for energy power supply, the development of technologies for long-distance transmission of energy power in direct current has been in the focus of R&D activities, which have addressed research in the area of higher-level voltage cables. Due to high DC voltages in close vicinity to the PE insulations of cables, however, space charges are accumulated, which alter the distribution of the electric field and produce local intensifications that accelerate the degradation processes of the insulations. A solution to counterbalance the occurrence of such phenomena is the introduction of PE-based nanocomposites as insulations of power cables; thermoplast polymer nanocomposites are a promising class of dielectrics as the addition of nanoparticles leads to the improvement of the polymers’ basic properties:

- While the introduction of microsized inorganic particles rarely results in an improvement of the electrical properties of composites, the reduction of the particle sizes to the order of nanometres results in improvements of the thermal, mechanical and electrical properties. This is very often due to the interface chemistry and interfacial strength, which increase exponentially in comparison to composites containing microsized particles.
- For the manufacturing of power cables, commonly insulating fillers such as layered silicates, montmorillonite clays, Al_2_O_3_, TiO_2_ and SiO_2_ are used; in the case of conducting fillers, graphite platelets and carbon nanotubes are among the most commonly used congeners. Such conducting fillers can improve the mechanical properties of nanocomposites (required in mechanical applications) and electrical conductivity (required for electromagnetic shielding and semiconductor layers of cables). With respect to insulating fillers, layered silicates or clays are in the range of a few nm in thickness and in the range of 100 nm in the other two dimensions, while the sizes of nanoparticles such as SiO_2_, TiO_2_ and Al_2_O_3_ are in the range of 30-40 nm.
- The introduction of low contents of nanofillers can increase the resistance to partial discharges (superficial and in the volume of the material), the resistance to electrical trees and water trees, the volume resistivity and the dielectric strength. The comparison of unfilled with nanofilled materials revealed that space charge accumulation phenomena were considerably affected by the presence of nanofillers.
- In the case of nanocomposites, the permittivity is often reduced, and, at low frequencies, it is found to be a ‘quasi-DC’ feature (large negative slopes in both the real permittivity and tan delta). By using compatibilizers, an increase of the permittivity can be obtained for certain values of the frequency. Depending on the type of compatibilizer, the nanocomposites lifetime can be increased over two orders of magnitude compared to the unfilled polymer.

Although PE-based nanocomposites offer many advantages for usage as insulators in power cables, some issues and short-comings remain to be solved, namely:

- the homogeneous dispersion of the nanoparticles and the characterization of the dispersion phase,
- the online control of the technological processes during commercial manufacturing of nanodielectrics,
- the detailed understanding of the interactions at the interfaces, the charge dynamics and the electrical breakdown behavior of such systems,
- studies of ageing and degradation mechanisms of nanodielectrics (required for their use as cable insulations in submarine applications, superconducting cables, etc.), and
- the detailed understanding of interactions at molecular/atomic level (e.g., the DC current decreases if certain types of nanofillers are added to PI and increases, on the other hand, if layered silicates are dispersed in EVA and PP).

The use of PE-based nanocomposites in industrial applications is also related to the volume of information regarding their behavior under the stress factors of operation. For example, little knowledge exists regarding the long-term toxicity of nanocomposites and the lifetimes of nanodielectrics. The surface treatment of the nanoscaled fillers is required in order to improve their compatibility with the polymer matrix: The filling grade, particle type, particle size and size distribution, aspect ratio and surface modification jointly alter the properties of the nanocomposites. In summary, the dielectric properties of PE-based nanocomposites show considerable improvements in comparison to unfilled polymers but on-going R&D activities must contribute to the detailed understanding of these phenomena. In order to use PE based nanocomposites for power cables insulation, the following strategies should be considered in the future:

- the mechanisms of doping nanoparticles for improving the characteristics and properties of insulation materials,
- the variations of the electrical conductivity and space charge under the combined action of heat and electrical field,
- the behavior of nanodielectrics to long-term applications in operation (i.e., lifetime estimation to multiple demands),
- the manufacture of recyclable materials for insulation.

## Figures and Tables

**Table 1:** Examples of different types of nanofillers classified by their origin (Adapted from reference [61]).

Origin		Selected Examples of Nanofillers
natural	animal	silk, wool, hair
	mineral	asbestos
	cellulose	wood, seed, leaf, fruit, stalk, bast
synthetic	inorganic	*oxides:* TiO_2_, SiO_2_, Al_2_O_3_, ZnO, MgO, Sb_2_O_3_
		*hydroxides:* Al(OH)_3_, Mg(OH)_2_
		*metals:* Al, Au, Ag, B, Sn, Cu, steel
		*silicates:* talc, mica, nanoclay, kaolin
		*salts:* CaCO_3_, BaSO_4_, CaSO_4_
		*carbides:* SiC *nitrides:* AlN, BN
	organic	*carbon based materials:* graphite fibers, nanotubes, carbon black, graphene
		*natural polymers:* cellulose and wood fibers, cotton, flax, starch
		*synthetic polymers:* aramid, polyester, polyamide, poly(vinyl alcohol) fibers

**Table 2:** DC relative volume resistivity of nanocomposites based on LDPE with 2 (or 5) wt % of different types of inorganic fillers. Adapted with permission by the author from reference [99].

Nanocomposite (Type of Filler)	Filler Content (wt %)	DC Relative Resistivity
Pure LDPE	0	1
LDPE/nano-SiO_2_	2	0.54
LDPE/nano-Al_2_O_3_	2	0.16
LDPE/nano-TiO_2_	2	1.07
LDPE/nano-TiO_2_	5	0.33

**Table 3:** Lengths of electrical trees depending on the content of MgO fillers in LDPE/MgO nanocomposite block specimens at 600 Hz. Adapted from reference [230].

MgO content (wt %)	0	1	2	5	8	102
Tree length (μm)	118	76	66	77	62	55

**Table 4:** Tree inception voltage determined by light emission in LDPE/MgO nanocomposites. Adapted from reference [231].

MgO content (wt %)	0	0.2	0.4	0.5	0.8	1
Tree Inception Voltage (kV_rms_)	2.65	2.73	2.93	3.09	3.13	3.16

**Table 5:** Breakdown strength of LDPE nanocomposites. Reprinted, with permission by the author, from reference [99].

Nanocomposites	Sample Thickness (mm)	*U_bd_* (kV)	*E_bd_* (kV·mm^−1^)	*A* (%)
Unfilled LDPE	0.51	23.304	45.694	4.42
LDPE/nano-SiO_2_–2 wt %	0.59	23.565	39.941	2.36
LDPE/nano-Al_2_O_3_–2 wt %	0.55	22.439	42.616	4.16
LDPE/nano-SiO_2_–5 wt %	0.55	21.820	39.673	12.46
LDPE/nano-Al_2_O_3_–5 wt %	0.53	21.273	40.138	8.37
LDPE/nano-TiO_2_–5 wt %	0.54	20.995	38.879	2.37

## Abbreviations

AC	alternating current
CB	carbon black
DC	direct current
ε*	relative complex dielectric permittivity
ε′/ε″	real/imaginary part of the complex relative permittivity
EHVC	extra high voltage cables
EPDM	ethylene-propylene-diene-monomer rubber
EPR/EPM	ethylene-propylene rubber
EVA	ethylene-vinyl-acetate
HDPE	high-density polyethylene
HV	high-voltage
HVAC	high-voltage alternating current
HVDC	high-voltage direct current
IEC	International Electrotechnical Commission
i-PP	isotactic polypropylene
LDPE	low-density polyethylene
LIPP	laser-induced pressure propagation method
LLDPE	linear low-density polyethylene
LV	low-voltage
MA	maleic anhydride
MMT	montmorillonite
MV	medium-voltage
PD	partial discharge
PE	polyethylene
PEA	pulsed electro-acoustic method
PI	polyimide
POSS	polyhedral oligomeric silsesquioxane
PP	polypropylene
tanδ	dissipation factor
TSM	thermal step method
XLPE	crosslinked polyethylene
